# Action control in emotion regulation: when reappraisal may carry hidden costs

**DOI:** 10.3389/fpsyg.2026.1790787

**Published:** 2026-04-30

**Authors:** David B. Rompilla, Connor Bazar, Savannah J. Dod, Valerie E. Lu, Takashi Yamauchi

**Affiliations:** Department of Psychological and Brain Sciences, Texas A&M University, College Station, TX, United States

**Keywords:** acceptance, action control, embodied cognition, emotion regulation, emotional memory, mouse tracking, reappraisal

## Abstract

**Introduction:**

Cognitive reappraisal is widely regarded as adaptive; however, strategies that impose control to reduce unpleasant emotion may involve tradeoffs when individuals encounter personally meaningful reminders of past adversity. The present study examined these dynamics within a cybernetic framework in which cognitive appraisal operates as a monitoring and corrective feedback process, whereas acceptance reflects disengagement from emotional control.

**Methods:**

Participants were undergraduates with a history of parental divorce (*n* = 33; M age = 20.09 years; M = 10.90 years since divorce), drawn from a larger experiment. They viewed sad film clips that included an unexpected divorce-relevant reminder, completed a movement-based Stroop task with mouse tracking to assess action dynamics, and later reported reminder-evoked distress. Habitual reappraisal and acceptance were measured via self-report.

**Results:**

Instructed reappraisal conferred short-term functional benefits, reflected in Stroop action dynamics indicating more decisive yet accurate responses during reminder exposure. Although greater time since divorce typically predicts lower reminder-evoked distress, moderation analyses showed that this association emerged only among individuals lower in habitual reappraisal and was attenuated at higher levels (time × reappraisal interaction, β = 0.08, *p* = 0.03). In contrast, higher habitual acceptance-operationalized as nonjudgment toward emotional experience-showed the expected pattern, with greater time since divorce predicting lower distress.

**Discussion:**

These findings highlight a potential tradeoff in emotion regulation: Reappraisal may support performance in the moment, yet greater habitual reliance on it may correspond with weaker links between time and reduced distress from past reminders. We interpret these effects as an exploratory proof-of-concept, suggesting that control-based regulatory processes may maintain emotional reference signals, whereas acceptance may facilitate their attenuation over time.

## Introduction

Imagine your parents separating during your childhood—a period in your life marked by emotional struggle, frequent arguments, and feeling caught in the middle. Years later, you sit down to watch a new movie, only to realize that its story centers on a family unraveling in a strikingly similar way. It has been years since that chapter of your life first unfolded—what do you feel in this moment?

Reminders of past adversity can arise unexpectedly through media, conversation, or memory, and the emotions they evoke may depend on how one habitually regulates emotion (Colombo et al., 2021). Some individuals tend to change their feelings in the face of distress through cognitive reappraisal—that is, by viewing unpleasant events in a different light. Others may instead rely on emotional acceptance, acknowledging their feelings without attempting to alter them. Both strategies are generally considered adaptive (Aldao et al., 2010; Ford et al., 2018; Gross and John, 2003; Kotsou et al., 2018; Naragon-Gainey et al., 2017), yet little is known about how they differentially shape one's ability to become less affected by reminders of past adverse events.

The present study examined this question using a naturally occurring subsample of individuals who had experienced parental divorce—a distressing yet common experience among college-aged adults (Johnston et al., 2025; Ross and Miller, 2009)—who were unexpectedly shown a sad film clip depicting a child undergoing parental divorce as part of a broader study (Rompilla et al., under review). We considered the possibility that habitual reappraisal, while often beneficial in the moment, may sustain emotional reactivity to such reminders by fostering continued efforts to control one's emotions, whereas habitual acceptance may promote reduced distress through openness to emotional experience.

### Emotional reminders of past adversity

Unexpected reminders of past adversity can trigger intense emotions even long after the event. For example, encountering a scene in a film that mirrors one's own prior loss or trauma may bring back distressing feelings years later (Lau-Zhu et al., 2018; Iyadurai et al., 2019). In general, the passage of time tends to diminish the emotional impact of a distressing life event—the folk wisdom that “time heals all wounds” reflects the reality that emotional reactions usually fade as one processes the experience (Bruehlman-Senecal and Ayduk, 2015). Indeed, by young adulthood, many individuals feel less upset by childhood adversities, such as parental divorce, a common experience (nearly one-third of American children experience parental divorce before adulthood; Johnston et al., 2025) that often elevates risk for psychological distress in youth (Hetherington and Stanley-Hagan, 1999; Obeid et al., 2021; Strohschein, 2005; Tullius et al., 2022). However, people vary widely in how quickly they “get over” such events (Lansford, 2009), and a key moderating factor may be how they habitually regulate their emotions when confronted with reminders of the past.

### Reappraisal and acceptance

Two strategies commonly studied in affective science are cognitive reappraisal and emotional acceptance (Troy et al., 2018; Rompilla et al., under review). Reappraisal involves reframing the meaning of an event to alter its emotional impact (Gross, 2015). For example, a child experiencing parental divorce might think, “*this is better this way,”* or “*this challenge has made me stronger.”* In contrast, acceptance entails allowing one's emotions to unfold without judgment or attempts to change them (Ford et al., 2018). A child in the same situation might instead think, “*my parents' divorce makes me feel sad at times, and that's normal and okay to feel.”*

These approaches are opposite in their cognitive mechanisms—reappraisal aims to control and change one's emotional state, whereas acceptance emphasizes allowing emotion to occur without interference. However, both strategies are generally considered adaptive and have been linked to a variety of better psychological health outcomes (Aldao et al., 2010; Gross and John, 2003; Ford et al., 2018; Kotsou et al., 2018). High habitual use of reappraisal is associated with lower depression and greater well-being in both cross-sectional (Garnefski and Kraaij, 2006; John and Gross, 2004) and longitudinal research (Kraaij et al., 2002). Likewise, habitual acceptance—most typically operationalized as nonjudgment toward emotional experience (see Ford et al., 2018)—is associated with better psychological health and wellbeing (Ford et al., 2018; Kashdan et al., 2006; Rompilla et al., 2023).

### Time to process vs. immediate relief

Despite these shared benefits, reappraisal and acceptance differ in their temporal profiles of effectiveness (see Shallcross et al., 2010). Reappraisal tends to yield immediate relief—experimental studies consistently show that reappraising emotion-inducing stimuli reduces unpleasant feelings, enhances positive emotion, and dampens physiological stress responses compared to no regulation or alternative strategies (Troy et al., 2018; Rompilla et al., 2022). Reappraisal has also been shown to prevent unpleasant feelings from disrupting ongoing tasks or obligations (e.g., Rompilla et al., 2025, 2026; Zhu et al., 2023).

Acceptance, in contrast, often leads to short-term discomfort but facilitates recovery later. Rather than suppressing or altering emotion, acceptance involves experiencing it fully, which can initially heighten distress (Campbell-Sills et al., 2006; Troy et al., 2018). Yet over time, this openness supports faster emotional recovery and resilience. Brief mindfulness or acceptance inductions, for example, often increase unpleasant feelings during exposure but reduce distress upon re-exposure or during recovery periods (Crosswell et al., 2017; Kral et al., 2018; Uusberg et al., 2016). However, when goal-directed action requires emotional change, excessive reliance on acceptance may reduce progress toward those goals.

Reappraisal and acceptance, therefore, move along different paths. Reappraisal can happen quickly and does not necessarily require fully feeling or sitting with the emotion. Acceptance, by contrast, involves allowing the emotion to be experienced more directly, which may deepen processing and support longer-term adaptation by gradually building tolerance for one's natural, initial feelings. These differing routes not only distinguish the strategies but may also involve meaningful tradeoffs. For example, relying too heavily on reappraisal may sometimes come at the cost of natural emotional integration (e.g., Ford and Troy, 2019). To better understand how these tradeoffs unfold, it is helpful to view emotion regulation through the lens of cybernetic control theories (Bramson et al., 2023; Carver, 2018; Tamir, 2021), which describe emotional change as a feedback process aimed at reducing the gap between one's current and desired emotional states.

### Emotion regulation as feedback control: a cybernetic view

**Figure 1:**
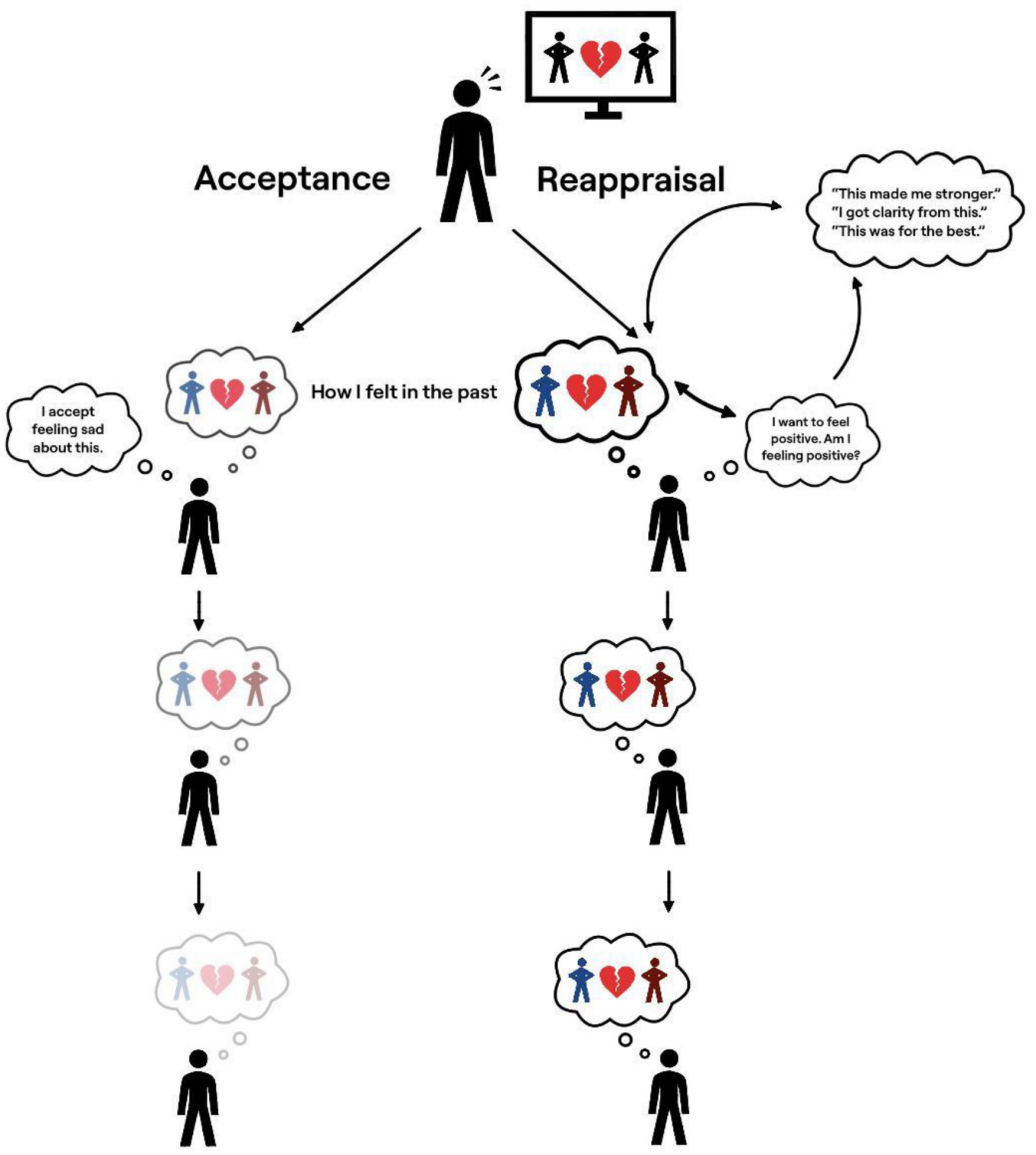
A cybernetic model of reappraisal and acceptance. Conceptual illustration of proposed cybernetic accounts of emotion regulation following exposure to a distressing reminder. The model depicts reappraisal as an evaluative process in which prior emotional experiences may be referenced when assessing whether current feelings align with a desired emotional goal state (e.g., “feeling positive”). In contrast, acceptance—operationalized here as a nonjudgmental stance toward emotional experience—is illustrated as reducing engagement with evaluative comparison processes. The figure is intended as a theoretical representation of possible mechanisms rather than a direct test of causal or longitudinal processes.

In cybernetic frameworks of self-regulation (Bramson et al., 2023; Carver, 2018; Tamir, 2021), emotions function as feedback signals within a negative feedback loop. Feelings are continuously compared to how a person wants or expects to feel, and mismatches between the two prompt regulatory action. For example, when someone is reminded of their parents' divorce and experiences sadness, that feeling is evaluated against an emotional goal, such as wanting to feel happy. Detecting a discrepancy (“I don't feel happy”) then initiates reappraisal (Bramson et al., 2023; Tamir, 2021) (see Figure 1).

Acceptance, however, aims to disengage from this evaluative process altogether. Rather than working to restore a preferred emotional state, acceptance reduces the importance of having an emotional goal (Garland et al., 2015; Teper and Inzlicht, 2013). Therefore, without a goal state, control mechanisms become less likely to be triggered (“I am sad, and that is okay”).

### When (too much) control may backfire

Because changing an emotion requires determining whether an emotional goal is reached, a reference of what it means to *not* be at that emotional goal is needed (e.g., Grisham et al., 2009; Carver and Scheier, 2001; see also Bramson et al., 2023). In other words, trying *not* to feel something requires recalling what it is like *to* feel it—a process that closely mirrors ironic mental control effects, such as the classic difficulty of trying not to think about a white elephant (Wegner, 1994; Wegner et al., 1993).

In terms of divorce reminders, memories related to how one does *not* want to feel in the face of a reminder helps them determine whether they need to regulate their emotions (see Bramson et al., 2023). Therefore, ironically, chronic attempts to control emotion that trigger reappraisal may also chronically bring past distress to mind (Tolin et al., 2002; see also Campbell-Sills et al., 2006; Chapman et al., 2013) (see Figure 1). Indeed, reappraisal has been shown to enhance autobiographical memory of unpleasant emotional events (Colombo et al., 2021; see also Wang et al., 2017).

In contrast, acceptance has been shown to *reduce* autobiographical memory of unpleasant emotional events (Colombo et al., 2024). By not engaging evaluative processes, the emotional system may become less likely to continually reactivate memories of past distressing feelings (Monachesi et al., 2023). Over time, these memories may weaken and eventually diminish (Eifert and Heffner, 2003; Ford et al., 2018; Wolgast et al., 2011; Leontyev and Yamauchi, 2025; Leontyev et al., 2019; Yamauchi and Xiao, 2018; Yamauchi et al., 2024, 2019; Leontyev and Yamauchi, 2021).

### The present study

The present study combined a cross-sectional examination of habitual emotion regulation with an experimental manipulation of instructed regulation within the same participants. Individuals who had experienced parental divorce viewed a sad film clip containing an unexpected divorce-relevant reminder while being instructed to reappraise, accept, or respond naturally. Habitual reappraisal and acceptance were assessed using self-report questionnaires, cognitive performance was measured using a motion-based Stroop task with mouse tracking, and reminder-evoked distress was assessed through retrospective self-report following the task. This design allowed us to examine both immediate functional effects of regulation and broader response patterns linked to habitual tendencies.

#### Habitual emotion regulation

Within this framework, we examined whether habitual use of emotion regulation strategies was linked to variation in a pattern often observed following major life events. In general, people further removed from experiences such as parental divorce may report less distress when reminded of them (Bruehlman-Senecal and Ayduk, 2015). Rather than assuming this pattern holds equally for everyone, we examined whether habitual reappraisal or acceptance related to how strongly time since divorce was tied to reminder-evoked distress.

Initial evidence from Shallcross et al. (2010) showed that individuals higher in habitual acceptance—which the habitual measure Shallcross et al. used operationalized as reduced avoidance of emotional experience—reported less unpleasant emotion during exposure to a sad, divorce-related film clip (i.e., the same film clip included in the present study), suggesting that letting go of control may shape responses to emotional reminders. However, little work has examined how these processes unfold when reminders are personally meaningful, and relatively few studies have considered whether habitual reappraisal might carry tradeoffs alongside its well-documented benefits (Ford and Troy, 2019).

Building on this work, we explored whether habitual reappraisal and acceptance were associated with different cross-sectional patterns linking time since divorce and reminder-evoked distress in a naturally occurring sample of individuals who had experienced parental divorce. Habitual reappraisal was operationalized as the tendency to regulate emotions by intentionally changing how one thinks about emotional situations, often with the goal of reducing unpleasant feelings or maintaining emotional composure (Gross and John, 2003). Emotional acceptance was operationalized as nonjudgment toward emotional experience (Ford et al., 2018; Baer et al., 2006), reflecting a non-evaluative stance toward one's emotions rather than broader operationalizations of acceptance that include willingness or reduced avoidance. This choice was made to provide a closer conceptual contrast with reappraisal, which inherently engages evaluative cognitive processes. Rather than testing directional hypotheses, our goal was to examine whether these regulatory tendencies were linked to stronger, weaker, or altered associations between time since divorce and emotional responses to the divorce reminder.

#### In-the-moment functional consequences of instructed emotion regulation

In addition to these habitual patterns, we also examined the immediate functional consequences of instructed emotion regulation in the same participants. In a complementary experimental component, participants were instructed to engage in reappraisal, acceptance, or no regulation while viewing the reminder, followed by assessments of cognitive and emotional performance. To evaluate short-term functional effects, cognitive performance was assessed using a motion-based Stroop task (i.e., a choice-reaching task), in which participants used the cursor to select the text color of a word while ignoring what the word said (e.g., “Red” shown in blue). Compared to traditional key-press methods, mouse movement more closely emulates natural forward action and provides sensitivity to conflict, hesitation, motor preparation, and subtle influences of emotional states (Freeman and Ambady, 2011; Spivey et al., 2005; Yamauchi and Wang, 2025).

Faster responses and earlier movement dynamics on this task—when not accompanied by accuracy costs—are thought to reflect more efficient decision making, while movement-based measures provide insight into how emotional states shape ongoing action. Guided by prior work (Rompilla et al., 2022, 2025, 2026; Zhu et al., 2023), we examined whether reappraisal instructions were associated with more efficient decision making on this task, while situating these in-the-moment effects alongside the cross-sectional patterns linked to habitual regulation.

## Methods

### Participants

As part of a broader study (Rompilla et al., 2026), 152 undergraduate psychology students were recruited from the Texas A&M University subject pool. From the initial sample, 34 students reported *after* completing all experimental procedures that they had experienced parental divorce. One participant did not report demographic information (i.e., age and gender) and had to be excluded from analyses, leaving a final sample of 33 students (Mage = 20.09 years, *SD* = 3.81; 72.73% female) who had experienced parental divorce and had all necessary measures. Participants provided informed consent, and all procedures were approved by the Texas A&M University Institutional Review Board (IRB2021-0908D).

No study predictions were preregistered. Analytic decisions were informed by an ongoing broader project (Rompilla et al., 2026), and analyses were planned while data collection was already underway. The decision not to prescreen participants for parental divorce history was intentional. By assessing divorce history only after task completion, the study preserved the experience of encountering a personally meaningful reminder unexpectedly within a controlled laboratory context. This design allowed us to examine emotion regulation in response to a laboratory-controlled yet spontaneous reminder of personal adversity—an approach that differs from many studies comparing reappraisal and acceptance, which often rely on fully anticipated, explicitly targeted emotional stimuli or on naturally occurring experiences assessed outside the laboratory that lack consistency in emotional content across participants.

Given these constraints and goals, the present study should be interpreted as an exploratory proof-of-concept. Rather than testing preregistered hypotheses, the study aimed to characterize response patterns associated with habitual and instructed reappraisal and acceptance in the presence of a personally meaningful emotion elicitor. All study materials, data, and experimental procedure files are publicly available (https://osf.io/as2tq/files).

Sample-level race and ethnicity data were not directly collected. However, departmental demographic statistics from the Texas A&M University Department of Psychological and Brain Sciences for the 2023 academic year indicate that the undergraduate population was approximately 43.9% White, 23.4% Hispanic, 19.7% Asian, 8.3% Black, and 4.7% “other” [Texas A&M University, 2025; a preserved version of these data is available at https://osf.io/as2tq/files].

A sensitivity power analysis was conducted in *G*Power 3.1.9.7 using an *F* test for multiple regression (fixed model, R^2^ increase; Faul et al., 2007). With an alpha level of 0.05, desired power (1 – β) of 0.80, and a total sample size of *n* = 33, the detectable effect size for a single tested predictor (with five total predictors in the model) was *f*^2^ = 0.26, corresponding to a medium-to-large effect (Cohen, 1988). Thus, the study was adequately powered (80%) to detect effects of moderate magnitude or greater in the moderation analysis, though not small effects.

The size of this subgroup is comparable to prior research isolating populations with specific adversity (e.g., *N* = 23 in Schweizer et al., 2016) and to studies examining emotion regulation mechanisms (e.g., *N* = 34 in Neta et al., 2023, Study 2). Notably, the current design offered a unique methodological advantage: participants were not asked about parental divorce until *after* the task, preventing expectancy effects and ensuring that the divorce-related reminder used during the study was experienced as genuinely spontaneous.

### Procedure

**Figure 2:**
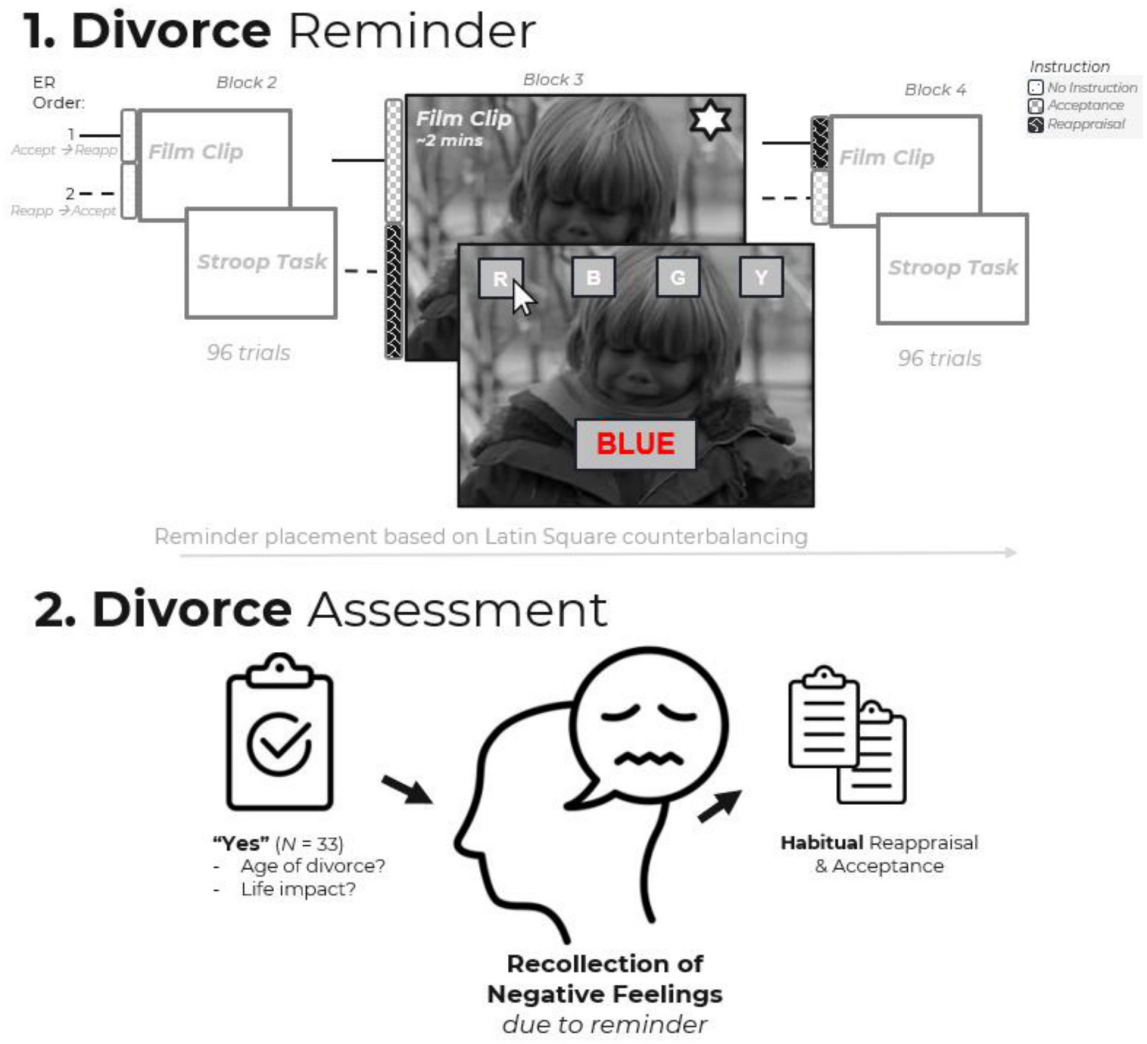
Experimental procedures. Participants completed four experimental blocks following practice trials. Each block consisted of a film clip, viewing instructions (no instruction, acceptance, or reappraisal), emotional self-reports, and a Stroop task. Block 1 (not shown) served as a neutral baseline. Blocks 2–4 featured sad film clips presented in counterbalanced order, one of which contained the divorce-relevant reminder. The reminder is depicted in Block 3 for illustrative purposes only; actual reminder placement varied across participants based on Latin square counterbalancing. The instruction under which the reminder was received and the block in which it occurred were controlled for in analyses. Following the task, participants reported parental divorce history, reminder-evoked negative feelings, and habitual emotion regulation tendencies.

Figure 2 provides an overview of the procedure (see Rompilla et al., 2026, for bigger-picture procedure). The study was completed in a quiet laboratory setting using PsychoPy (v 2023.2.3) (Peirce, 2007). Upon arrival and informed consent, participants provided demographic information (i.e., gender and age) and were then introduced to a Stroop task, given instructions, and completed six practice trials with computerized feedback. Participants then completed four experimental blocks. Each block consisted of a film clip, instructions for how to view the clip, emotional self-reports, and a Stroop task following the clip. The structure of these blocks was the same, varying only in the instruction given, film clip, and measures specific to emotion regulation (reappraisal or acceptance).

Block 1 served as a baseline, featuring a neutral film clip. This block established a comparison point for emotional reactivity (to verify that the sad clips successfully induced the intended emotion; see Rompilla et al., 2026) and also provided additional Stroop task practice before the critical experimental blocks. Blocks 2–Block 4 were experimental blocks that involved three different sad film clips, with the divorce reminder being one of those film clips. Therefore, the block where the reminder occurred was random and based on assignment to different film clip orders (determined by Latin square counterbalancing). Where participants received the divorce reminder and the instructions under which they received the reminder were controlled for in analyses. After all blocks, participants reported whether they had experienced parental divorce and, if so, the age at which their parents separated and whether the divorce reminder brought back unpleasant feelings from that experience. They then completed habitual reappraisal and acceptance measures.

### Measures

#### Divorce reminder

The divorce reminder was a clip (1 min 59 s) from the film *Kramer vs. Kramer*. This film clip involved a father discussing new divorce arrangements with their child, with the child being upset and crying because of the arrangements. Other sad film clips and the neutral baseline film clip included in the study did not involve divorce themes and were of comparable lengths (~2 mins; see Rompilla et al., 2026). All film clips were taken from Troy et al. (2018).

#### Divorce experience and distress brought back due to a reminder

##### Divorce experience

Participants were asked, “Have your parents ever separated or gone through a divorce?” (Yes, No). If they responded *Yes*, they were included in the sample.

##### Divorce distance

After responding yes, participants were asked, “How old were you when this happened?”. Divorce distance was then calculated by subtracting the divorce age from the participant's current age (recorded from the demographics).

#### Divorce-related recollections

After all study blocks, participants were specifically asked how much the divorce reminder in the study brought back negative feelings relevant to their experience. This question read as follows: “How much did the film clips in the study bring back negative feelings from past parental separation or divorce?” (0 = “Not at all”, 1 = “Faint”, 2 = “Some”, 3 = “Vivid”, 4 = “Intense”).

### Habitual reappraisal and acceptance

#### Reappraisal

The Reappraisal subscale of Gross and John's (2003) Emotion Regulation Questionnaire was used to measure habitual reappraisal. Participants completed eight items (e.g., “When I am faced with a stressful situation, I make myself think about it in a way that helps me stay calm.”, “I control my emotions by changing the way I think about the situation I'm in.”, etc.) on a scale of 1 = Strongly Disagree-−7 = Strongly Agree (Prompt: *Please rate how much you agree with the below statement*). Higher scores on all items indicated higher habitual reappraisal. Internal consistency for this scale was good (α = 0.82).

#### Acceptance

The Nonjudgment subscale of the Five Facet Mindfulness Questionnaire (Baer et al., 2006) was used to measure habitual acceptance. Participants completed six items (e.g., “I tell myself I shouldn't be feeling the way I'm feeling.”, “I think some of my emotions are bad or inappropriate and I shouldn't feel them”, etc.) on a scale of 1 = Never true-−5 = Always true (Prompt: *Please rate each of the following statements with the number that best describes your own opinion of what is generally true for you*). All items were reverse-scored so that higher scores indicated higher acceptance. Internal consistency for this scale was very good (α = 0.90).

### Instructed regulation performance: strategy effectiveness in the moment

The subsample of participants who experienced parental divorce received the divorce reminder under one of three experimental conditions: Reappraisal (*n* = 11), acceptance (*n* = 8), or no Instruction (*n* = 14). Thus, we also measured the “in-the-moment” impact of this instructed regulation on subsequent cognitive performance and general emotional experience immediately following the film clip. Those under reappraisal instructions were instructed to “think about the situation you see in a more positive light,” while those under acceptance instructions were instructed to “allow yourself to experience your feelings without trying to change them.” Those given no instructions were told to “view the film clip as you naturally would.” These instructions were adapted from Troy et al. (2018); see Supplementary Appendix A for full instructions). Reappraisal and acceptance instructions were delivered via audio and could not be skipped. At the end of Block 3, participants provided typed descriptions of how they applied the assigned strategy, a requirement they had been informed of in advance. This adaptation was intended to ensure that participants actively engaged with the strategy instructions.

#### Cognitive performance

##### Stroop task

Participants completed 96 trials of a choice-reaching, mouse-cursor-based Stroop task following the divorce reminder (see Supplementary Figure S1 and Supplementary Appendix B for more task details; Rompilla et al., 2026). On each trial, a color word (e.g., “Red”) appeared in a font color that either matched (e.g., “Red” in red ink) or conflicted with (e.g., “Red” in blue ink) its meaning. Participants were instructed to click the button corresponding to the font color, not the word meaning. Four response buttons were displayed across the top of the screen and labeled with the first letters of each color: “R,” “B,” “G,” and “Y” (i.e., Red, Blue, Green, Yellow). The order of these buttons remained fixed across blocks. Each trial followed a consistent sequence: a 1-s fixation period, a 0.5-s target display, cursor-based response selection, and a 0.5-s progress bar before the next trial (see Supplementary Figure S1). To preserve emotional context, the Stroop tasks were presented over still images from the divorce reminder. These images progressed sequentially across the 96 trials (see Supplementary Figure S1).

All trials that involved other film clips that were not the divorce reminder followed this same format. Differences in the block in which the reminder was received were accounted for in all analyses.

##### Measures

Effectiveness was defined by response speed, time of maximum velocity, time of maximum acceleration, and accuracy. Following the recommended procedures of Spivey et al. (2005), mouse-trajectory analysis was used to calculate these metrics (see Supplementary Appendix B for more details). *Response time* measured how quickly participants responded (time from stimulus onset to button press). *Time of maximum velocity* and *time of maximum acceleration* indexed the points at which cursor speed and acceleration peaked, computed from the trajectory's velocity profile. *Accuracy* reflected whether the correct response was selected. Faster response times, along with earlier times of maximum velocities and acceleration, were thought to index more efficient cognitive decisions in the face of emotional distress.

#### General emotional experience

Immediately following the divorce reminder, participants reported valence and arousal ratings, and then viewed an 8-second countdown overlaid on the final frame of the video before beginning the Stroop task. Emotional self-reports of valence and arousal were collected following each video. Ratings were made on a slider scale ranging from −4 to +4 (valence: −4 = *negative*, 0 = *neutral*, 4 = *positive*; arousal: −4 = *down*, 0 = *neutral*, 4 = *stirred up*). Each scale was accompanied by expressive face icons (e.g., frowny face, happy face, depressed-looking face, alert-looking face) to reinforce interpretation (Betella and Verschure, 2016). At the end of a given block, participants reported how much sadness and happiness they felt while watching the film clip they watched during the block (1 = *not at all*, 9 = *an extreme amount*).

### Analysis plan

Analyses focused on two complementary questions concerning in-the-moment functional responses and cross-sectional patterns linked to habitual emotion regulation. First, we examined whether instructed reappraisal, acceptance, and no-instruction conditions were associated with differences in Stroop task performance and immediate emotional responses following the divorce reminder. Second, we evaluated whether habitual reappraisal and acceptance were related to variation in the association between divorce distance (i.e., time since divorce) and reminder-evoked distress.

To examine in-the-moment functional responses, we conducted a series of between-subjects ANCOVAs comparing three instruction conditions (reappraisal, acceptance, and no instruction) on Stroop task performance (response time, idle time, time of maximum velocity, time of maximum acceleration, and accuracy) and on immediate emotional experience (valence, arousal, sadness, and happiness) following the divorce reminder. All ANCOVAs controlled for gender and the block in which the reminder occurred (Block 2, 3, or 4). Tukey post hoc tests were used to probe specific group differences. As a robustness check, we conducted supplementary linear mixed-effects models that preserved all trial-level observations (rather than collapsing across the 96 trials within each block) and included random intercepts for participants to account for within-subject variability and the small, uneven group sizes inherent in the between-subjects design.

To examine cross-sectional patterns linked to habitual emotion regulation, we conducted two separate moderation models—one with habitual reappraisal and one with habitual acceptance—using R-based code that reproduced PROCESS Model 1 (Hayes, 2022). Each model controlled for gender and the instruction condition under which the divorce reminder occurred (reappraisal, acceptance, or no instruction). For significant interactions, we examined simple slopes of divorce distance at low (−1 *SD*), mean, and high (+1 *SD*) levels of the moderator. To evaluate robustness, models were re-estimated using HC3 robust standard errors to reduce sensitivity to unequal variance or influential observations. Johnson–Neyman analyses were conducted to identify values of the moderator at which divorce distance significantly predicted reminder-evoked distress.

## Results

### Preliminary descriptives and divorce time-recollection relationship

On average, participants reported that the divorce reminder brought back at least “some” (i.e., *M* = 2.12, *SD* = 1.24) negative feelings from their experience with parental divorce, confirming the effectiveness of the reminder. Personally relevant distress from the reminder was also uncorrelated with all general emotional response measures following the film clips (valence, arousal, sadness, and happiness; *p*s > 0.54; see Supplementary Table S1 for zero-order Pearson correlations across all study variables), underscoring the importance of treating our measure of distress specifically elicited by personal relevance as a distinct construct. Participants were distributed across a broad range of time since divorce (*M* = 10.90 years ago, *SD* = 5.80) with representation from relatively recent to distant divorces (min = 2 years ago, max = 21 years ago; see Supplementary Figure S2 for full distribution). Initial Pearson correlation patterned in the expected direction, where greater time since divorce was associated with lower intensity of divorce-related recollections, although this association did not quite reach significance (*r* = −0.33, *p* = 0.065).

### Short-term effects of emotion regulation instruction

#### Stroop performance

Participants randomly fell into one of three instruction conditions under which they experienced the divorce reminder: reappraisal (*n* = 11), acceptance (*n* = 8), or no instruction (*n* = 14). Key variables were comparable and not significantly different across groups, including age, divorce distance, habitual reappraisal, and habitual acceptance (see Supplementary Table S2). The no instruction condition contained a higher proportion of males (42.9%; male *n* = 6, female = 8) than other conditions. However, acceptance (12.1%; male *n* = 1; female = 7) and reappraisal (18.2%; male *n* = 2; female = 9) proportions were close to equivalent. Accuracy was uniformly high with minimal variability across participants (*M* = 99.5%, *SD* = 1.5%). Furthermore, no significant effects of instruction were observed for accuracy (all *p*s > 0.05; see *F*-test below), and accuracy was uncorrelated with all other study variables (all *p*s > 0.05; see Supplementary Table S1). Thus, observed differences in performance were likely not attributable to accuracy tradeoffs.

A series of between-subjects ANCOVAs examined whether instruction during the divorce reminder (reappraisal, acceptance, or no instruction) influenced Stroop task performance following the reminder, controlling for gender and the block in which the reminder occurred (Block 2, 3, or 4). To account for individual differences in baseline performance, Stroop performance following the neutral baseline (Block 1) was subtracted from performance following the divorce reminder.

**Figure 3:**
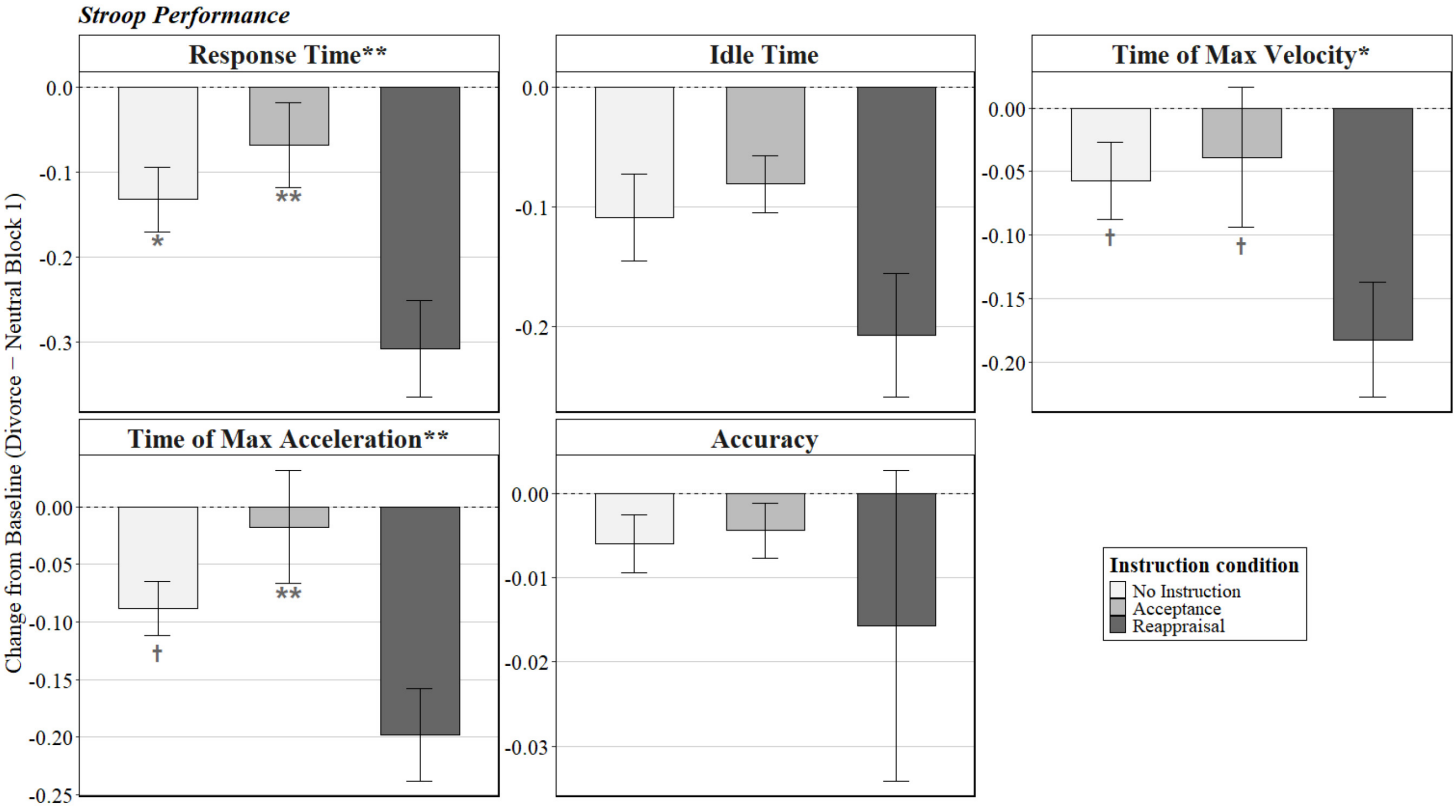
Short-term functional benefits of reappraisal. Bars represent change scores calculated by subtracting baseline performance in Block 1 (neutral film) from performance during the divorce-reminder block. Negative values indicate faster or earlier responding relative to baseline. Error bars represent ±1 SE. Asterisks in panel titles indicate a significant main effect of instruction condition. Symbols above individual bars denote Tukey post hoc comparisons with those who received the reminder during reappraisal (**p < 0.01, *p < 0.05, ^†^*p* < 0.10). All other pairwise comparisons were nonsignificant.

For response time, the effect of instruction was significant, *F*_(2, 28)_ = 7.15, *p* = 0.003, partial η^2^ = 0.34 (Figure 3). Tukey post hoc tests indicated that participants who viewed the reminder under reappraisal instruction (*M* = −0.32, *SD* = 0.21) responded significantly faster than those instructed to accept their emotions (*M* = −0.07, *SD* = 0.15) or given no instruction (*M* = −0.13, *SD* = 0.15).

The effect of instruction was also significant for time of maximum velocity, *F*_(2, 28)_ = 4.77, *p* = 0.016, partial η^2^ = 0.25, and for time of maximum acceleration, *F*_(2, 28)_ = 6.52, *p* = 0.005, partial η^2^ = 0.32. Tukey post hoc comparisons mirrored the response time results, such that reappraisal instruction was associated with earlier times of maximum velocity and acceleration (see Supplementary Table S3 for means and standard deviations). However, only the comparison between reappraisal and acceptance for time of maximum acceleration reached significance (*p* < 0.01), with other comparisons between reappraisal and acceptance and no-instruction conditions trending toward significance (*ps* < 0.10; see Figure 3).

The effect of instruction was not significant for idle time, *F*_(2, 28)_ = 2.14, *p* =0.14, partial η^2^ =0.13, nor for accuracy, *F*_(2, 28)_ = 0.93, *p* = 0.41, partial η^2^ = 0.06.

##### Robustness

To address concerns related to the small and uneven group sizes inherent in the between-subjects design, we conducted supplementary analyses using mixed-effects models in R. Linear mixed-effects models were estimated using the lmer() function from the *lme4* package for continuous outcomes, and generalized mixed-effects models with a binomial distribution and logit link were estimated for accuracy. Models were specified as:

*DV* ~ *Strategy* × *Congruency* + *baseline covariate* + *Gender* + *DivExposure_Block* + *(1 | Participants)*.

These models were fit at the trial level and included random intercepts for participants, allowing for more efficient use of the available data by accounting for within-subject variability. Separate models were estimated for response time, time of maximum velocity, time of maximum acceleration, idle time, and accuracy. For the continuous dependent variables (response time, time of maximum velocity, time of maximum acceleration, and idle time), values were log-transformed prior to analysis to reduce skewness; when necessary, a constant was added prior to transformation to accommodate zero or negative values. Accuracy, which was binary at the trial level, was analyzed using a binomial-logit mixed-effects model, with baseline accuracy represented using an empirical logit transformation of Block 1 accuracy.

Each model included fixed effects of strategy (reappraisal, no instruction, acceptance), congruency (congruent, incongruent), and their interaction. To account for pre-existing individual differences in task performance, the corresponding Block 1 baseline measure for each dependent variable was included as a grand-mean centered covariate. Gender and the block in which the divorce reminder occurred were also included as covariates.

Models were fit using maximum likelihood estimation (REML = FALSE for linear models), and fixed effects were evaluated using Type III tests via the *lmerTest* package. Follow-up comparisons were conducted using estimated marginal means (*emmeans*) with Holm correction.

Across all dependent variables, the pattern of results was consistent with those obtained from the primary ANCOVA analyses. In particular, effects of strategy and congruency remained in the same direction and of comparable magnitude, and no new interactions emerged. These findings suggest that the primary results are robust to analytic approach and are not driven by aggregation of the data into between-subject summaries. Full model results are reported in Supplementary Table S4 in the Supplementary material.

##### Summary: short-term functional benefits of reappraisal

Overall, instructed reappraisal conferred short-term functional benefits: participants told to reappraise the divorce reminder showed faster and earlier decision making during the Stroop task, without corresponding costs to performance accuracy (accuracy was uniformly high: *M* = 99.5%, *SD* = 1.5%).

#### Emotional experience

Another series of between-subjects ANCOVAs examined whether instruction during the divorce reminder (reappraisal, acceptance, or no instruction) influenced participants' emotional experience (valence, arousal, sadness, and happiness) reported immediately after the reminder, again controlling for gender and the block in which exposure occurred (Block 2, 3, or 4). To account for individual differences in baseline emotional responding, emotional experience reported following the neutral baseline (Block 1) was subtracted from reports following the divorce reminder. Across all four emotional measures—valence, arousal, sadness, and happiness—the effect of instruction condition was nonsignificant. All *F* values were below 1.40, and all *p-*values exceeded 0.26, indicating that participants reported comparable levels of emotional experience across conditions (see Supplementary Table S5 for full results; Supplementary Figure S3 for plots of means).

##### Summary: no short-term effects on emotional experience

In contrast to Stroop performance, reappraisal did not confer short-term functional benefits in terms of reported emotional experience in the moment.

### Longer-term effects of habitual emotion regulation on time since divorce

**Figure 4:**
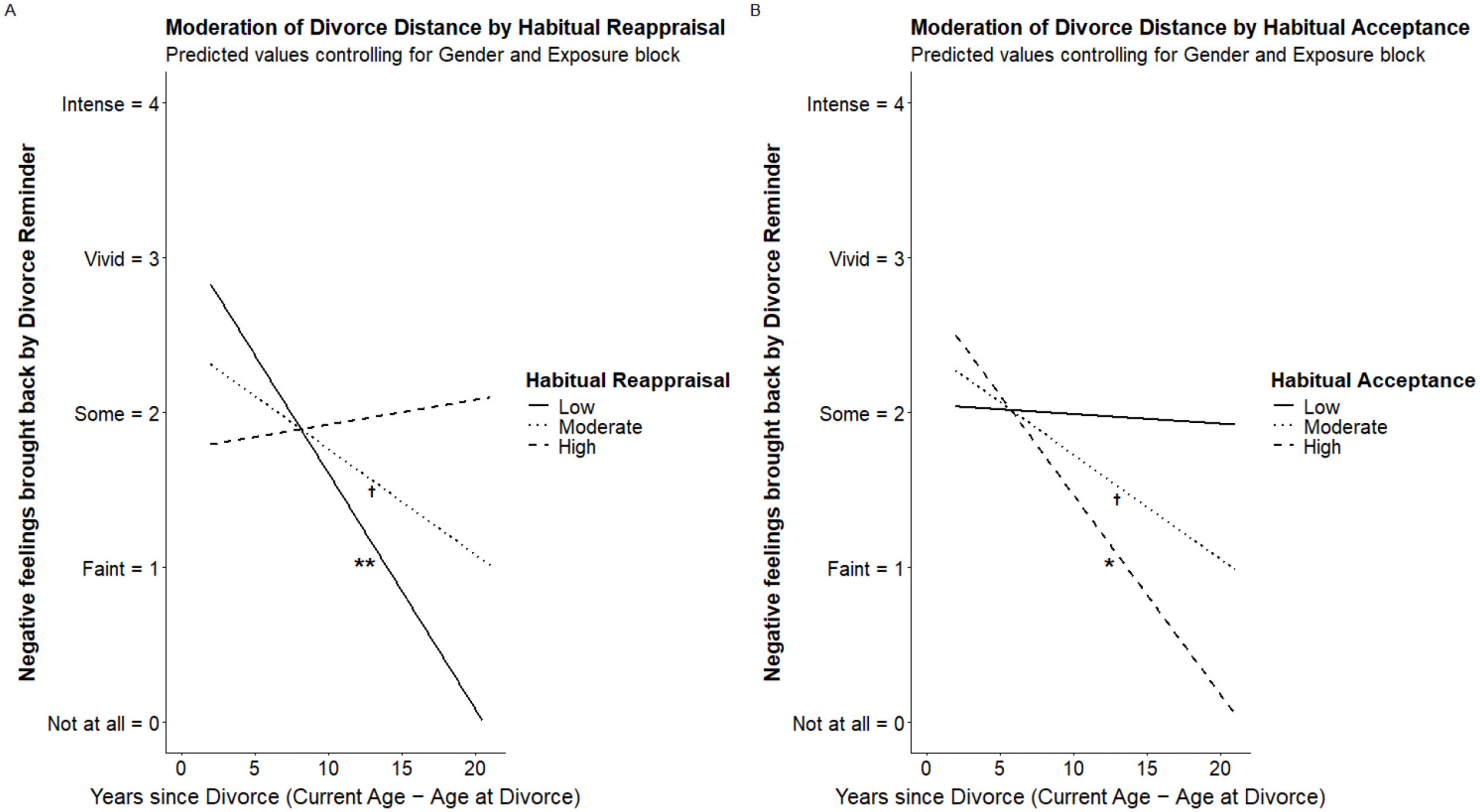
Longer-term effects of habitual emotion regulation: simple slopes. Panels display predicted values from moderation models examining whether habitual reappraisal **(Panel A)** and habitual acceptance **(Panel B)** moderated the association between years since parental divorce and negative feelings elicited by the divorce reminder. Predicted values are plotted at low (−1 SD), moderate (mean), and high (+1 SD) levels of the moderator while controlling for gender and the block in which the reminder occurred. The y-axis reflects self-reported intensity of negative feelings brought back by the reminder (0 = not at all, 4 = intense). Symbols indicate simple slope tests (**p < 0.01, *p < 0.05,^†^*p* < 0.10).

**Figure 5:**
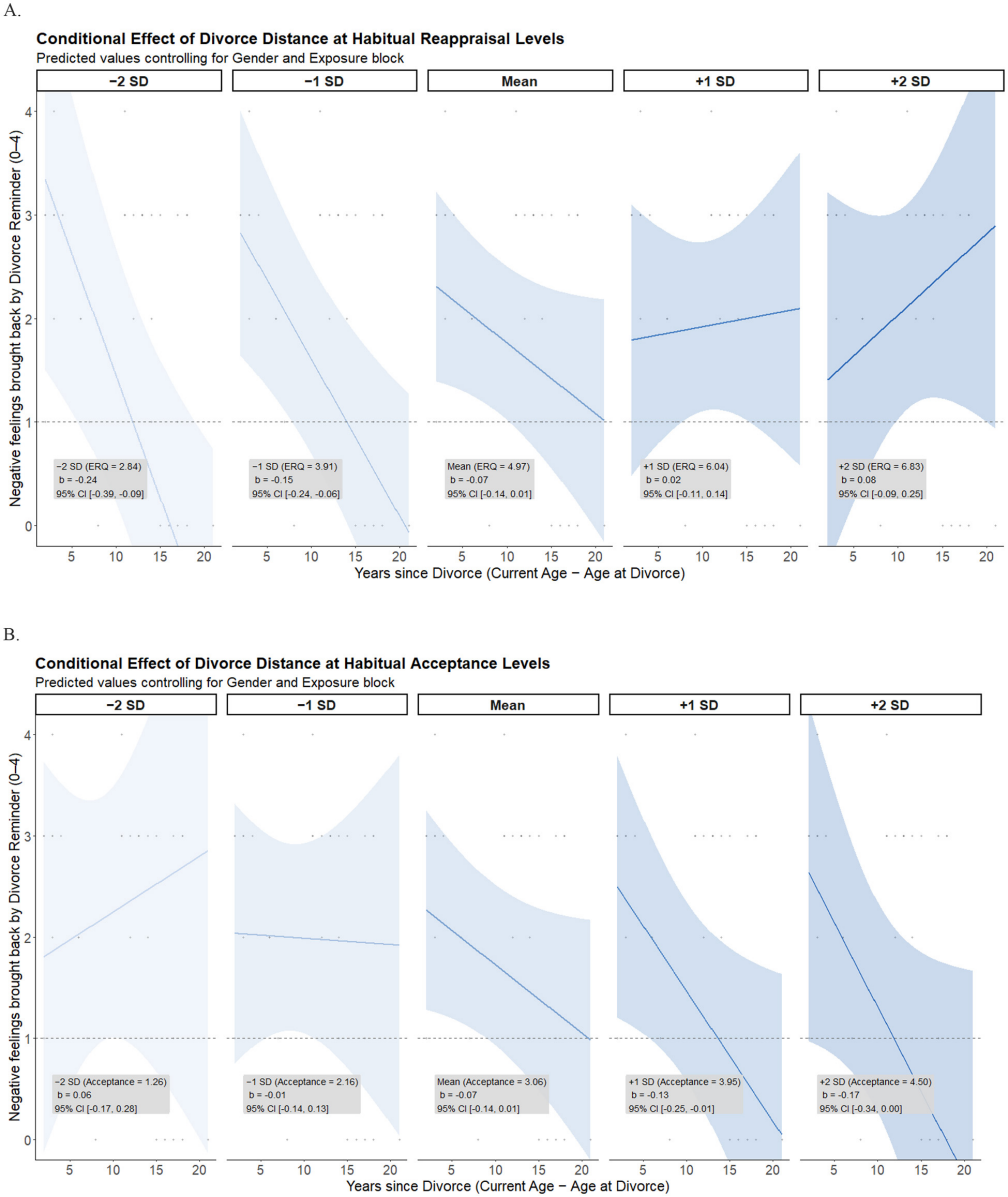
Longer-term effects of habitual emotion regulation: conditional effects. Panels display conditional effects of years since parental divorce on negative feelings elicited by the divorce reminder at discrete levels of habitual reappraisal **(A)** and habitual acceptance **(B)**. Predicted values are shown at −2 SD, −1 SD, the mean, +1 SD, and +2 SD of the moderator while controlling for gender and the block in which the reminder occurred. Shaded regions represent 95% confidence intervals. The y-axis reflects self-reported intensity of negative feelings brought back by the reminder (0 = not at all, 4 = intense).

To examine cross-sectional patterns linked to habitual regulation, we evaluated whether the association between time since parental divorce and intensity of divorce-related distress brought back by the reminder was moderated by habitual reappraisal and acceptance, controlling for gender and the instruction under which divorce exposure occurred. Significant moderation was indicated by significant habitual reappraisal x divorce distance or habitual acceptance x divorce distance interaction effects. Figure 4 shows simple slopes effects for these interactions, and Figure 5 shows conditional effects. Supplementary Tables S6, S7 show the full output for each moderation model. Supplementary Table S1 includes zero-order Pearson correlations across all study variables.

#### Habitual reappraisal as moderator

A regression model including divorce distance, habitual reappraisal, their interaction, and the covariates (gender and instruction during exposure) was significant, *F*_(6, 26)_ = 2.66, *p* = 0.038, *R*^2^ = 0.38 (see Supplementary Table S6). In support of our hypothesis, the divorce distance × habitual reappraisal interaction was significant, Δ*R*^2^ =0.126, *F*_(1, 26)_ = 5.27; β = 0.08, *SE* = 0.05, *t* = 2.30, *p* = 0.030, 95% CI (0.01, 0.15).

Simple slopes indicated that *greater* distance from the divorce predicted significantly lower recollected distress at *low* levels of reappraisal (−1 *SD*; <3.90), β = −0.15, *SE* =0.05, *t* = −3.42, *p* = 0.002, 95% CI (−0.24, −0.06). The effect was nonsignificant for reappraisal at the mean (i.e., 0; *M before normalization* = 4.97), β = −0.07, *SE* = 0.04, *t* = −1.83, *p* = 0.079, 95% CI [−0.15, 0.01], and at high levels of reappraisal (+1 *SD*; > 6.04), β = 0.02, *SE* = 0.06, *t* = 0.27, *p* = 0.788, 95% CI (−0.11, 0.14) (Figures 4, 5).

##### Robustness and specificity

HC3-robust estimates yielded the same results, with the interaction effect remaining nearly identical, β = 0.08, *SE* = 0.03, *t* = 2.31, *p* = 0.029, 95% CI (0.01, 0.15). These findings indicate that the results were robust and not driven by heteroscedasticity or outliers.

In addition, Johnson–Neyman analysis indicated that greater time since divorce significantly predicted lower reminder-evoked distress for individuals reporting habitual reappraisal scores between 2.83 and 4.89 on the 1–7 scale (*M* = 4.97) (see Supplementary Figure S4). Thus, the association between divorce distance and reduced distress was evident for individuals with *low to slightly below-average* levels of habitual reappraisal, but not for those at or above the sample mean.

##### Summary: preserved reminder-evoked distress under habitual reappraisal

Overall, the association between greater time since divorce and lower reminder-evoked distress was evident primarily among individuals reporting lower levels of habitual reappraisal. This normative pattern was not observed at average or higher levels of reappraisal, where divorce distance was unrelated to reminder-evoked distress (see Figures 4, 5). Thus, rather than indicating that reappraisal uniformly maintains distress, the findings suggest that the expected cross-sectional link between time and reduced distress was attenuated as habitual reliance on reappraisal *increased*.

#### Habitual acceptance as moderator

Regression including divorce distance, habitual acceptance, their interaction, and the covariates (gender and instruction during exposure) was not significant, *F*_(6, 26)_ = 1.64, *p* = 0.177, *R*^2^ = 0.27 (Supplementary Table S7), and, the divorce distance × habitual acceptance interaction was not significant, Δ*R*^2^ = 0.04, *F*_(1, 26)_ = 1.59; β = −0.07, *SE* =0.05, *t* = −1.26, *p* = 0.219, 95% CI (−0.18, 0.04).

Opposite to reappraisal, greater distance predicted significantly lower distress at *high* levels of acceptance (+1 *SD;* > 3.96), β = −0.13, *SE* =0.06, *t* = −2.23, *p* =0.035, 95% CI (−0.25, −0.01). At mean levels of acceptance (i.e., 0; *M before normalization* = 3.06), β = −0.07, *SE* =0.04, *t* = −1.80, *p* = 0.084, 95% CI (−0.14, 0.01), and low levels of acceptance (−1 *SD*; <2.16), β = −0.01, *SE* = 0.07, *t* = −0.10, *p* = 0.925, 95% CI (−0.14, 0.13), divorce distance became nonsignificant, and progressively less predictive of distress (Figures 4, 5).

##### Robustness and specificity

HC3-robust estimates yielded the same results, with the interaction effect remaining non-significant, β = −0.07, *SE* =0.08, *t* = −0.89, *p* = 0.384, 95% CI (−0.23, 0.09). These findings indicate that the results were robust and not driven by heteroscedasticity or outliers.

In addition, Johnson–Neyman analysis indicated that greater time since divorce significantly predicted lower reminder-evoked distress for individuals reporting habitual acceptance scores between 3.20 and 4.37 on the 1–5 scale (*M* = 3.06) (see Supplementary Figure S5). Thus, the association between divorce distance and reduced distress was evident for individuals with *high to slightly above-average* levels of habitual acceptance, but not for those at or above the sample mean.

##### Summary: descriptive pattern opposite to reappraisal

Overall, the interaction between habitual acceptance and divorce distance did not reach statistical significance. Nevertheless, the pattern of simple slopes differed descriptively from that observed for reappraisal. Whereas, divorce distance predicted lower reminder-evoked distress primarily at low levels of reappraisal, the association emerged at *higher* levels of acceptance. The acceptance patterns should be interpreted cautiously given the absence of a significant interaction, but they suggest that acceptance may relate to a different profile of responses across divorce distance (see Figures 4, 5).

#### Further diagnostics of moderation models

Given the small sample size, additional diagnostics were conducted for both moderation analyses to ensure that results were not driven by outliers or violations of model assumptions. These diagnostics revealed no concerning outliers, with the largest Cook's distance values (0.24 and 0.23) well below conventional thresholds (i.e., Cook's distances > 1.0). Residual, Q–Q, and scale–location plots likewise showed no evidence of assumption violations (see Supplementary Figure S6, S7).

## Discussion

Reminders of past adversity rarely arrive with warning. A scene in a film, a line of dialogue, or a fleeting image can suddenly reopen emotional chapters many believe they have already closed. The present study examined how responses to such reminders vary not only with time since the event, but also with habitual emotion regulation. In a naturally occurring subsample of undergraduate students with a history of parental divorce, instructed reappraisal was associated with faster and more decisive action-based responses during a cognitive task involving the reminder, suggesting adaptive benefits in the moment. At the habitual level, a different pattern emerged. Greater time since divorce was associated with lower reminder-evoked distress primarily among individuals *lower* in habitual reappraisal—operationalized here as the tendency to regulate emotion by intentionally changing how one thinks about emotional situations to reduce unpleasant feelings or maintain composure (Gross and John, 2003). Emotional acceptance—operationalized as nonjudgment toward emotional experience (Ford et al., 2018; Baer et al., 2006), reflecting a more non-evaluative stance toward emotion—showed a complementary descriptive pattern in which the association between time since divorce and distress was more evident at *higher* levels of acceptance.

Taken together, these findings point to two distinct pathways: evaluative forms of regulation may support efficient goal-directed action in the moment, whereas a less evaluative stance toward emotion relates differently to how distress corresponds with time since adversity. Importantly, given the cross-sectional nature of the moderation analyses and the absence of preregistration, these results are best interpreted as an exploratory proof-of-concept intended to generate future confirmatory work rather than to establish definitive causal conclusions.

### Control helps action—but relates differently to how distress corresponds with time

Consistent with prior work, reappraisal conferred clear short-term functional benefits (Rompilla et al., 2025, 2026; Zhu et al., 2023). When instructed to reappraise a personally meaningful divorce reminder, participants responded faster on a subsequent Stroop task and exhibited earlier movement dynamics—reaching peak velocity and acceleration sooner—than those instructed to accept their emotions or given no instruction, while maintaining comparable accuracy.

At the habitual level, however, a different pattern emerged that was not captured by momentary task performance. Greater time since divorce was associated with lower reminder-evoked distress primarily among individuals *lower* in habitual reappraisal, whereas this association was attenuated at higher levels of reappraisal. Descriptively, simple-slopes patterns for acceptance showed the opposite direction, with the association between time since divorce and distress appearing more evident at *higher* levels of acceptance, although the overall moderation effect was not statistically significant.

Taken together, these findings suggest that evaluative forms of regulation may support efficient action under emotional strain, while relating differently to how distress corresponds with time. From a mechanistic perspective, reappraisal's reliance on ongoing evaluation and adjustment may coincide with continued engagement with emotionally relevant material.

### Reappraisal as action control

Cybernetic and comparator-based models of emotion regulation (Bramson et al., 2023; Carver, 2018; Tamir, 2021) describe emotional change as a feedback process involving reference signals, error monitoring, and corrective adjustments—mechanisms rooted in foundational theories of movement and action control (e.g., Adams, 1971; Wolpert and Ghahramani, 2000). Reappraisal fits naturally within this architecture. To evaluate whether an emotional goal has been reached, the system must repeatedly reference the very emotional state it aims to change. Much like error correction in motor control, this process may enhance immediate performance while also increasing the likelihood that unwanted emotional content remains cognitively accessible (Wegner, 1994; Wegner et al., 1993). Importantly, this account does not imply that negation or inhibitory language is inherently maladaptive. Contemporary work suggests that linguistic negation can recruit inhibitory control processes that support adaptive emotional distancing (e.g., “I am aware of my sadness, but I am not my sadness”), indicating that ironic effects may depend on how regulatory control is engaged rather than on negation itself (Tettamanti et al., 2008; Beltrán et al., 2021).

Consistent with a cybernetic and comparator-based perspective, prior work shows that reappraisal can *enhance* memory for unpleasant emotional events (Colombo et al., 2021; Wang et al., 2017). Rather than implying that reappraisal maintains distress, this literature suggests that evaluative control processes may sometimes keep emotional information cognitively active. Within the present cross-sectional findings, this framework offers one possible interpretation for why associations between time since divorce and reminder-evoked distress differed across levels of habitual reappraisal. Acceptance—particularly the disengagement from evaluative control emphasized here—has shown the opposite pattern in some contexts, including *reduced* memory for unpleasant emotional events (Colombo et al., 2024), and may operate outside the comparator loop required for reappraisal (Eifert and Heffner, 2003; Ford et al., 2018; Monachesi et al., 2023). By reducing the need to reach a specific emotional goal, acceptance may allow emotional systems to reorganize without continuous corrective input (Crosswell et al., 2017; Kral et al., 2018; Uusberg et al., 2016).

#### When letting go may support emotional recovery

Together with prior work, the present findings point to two broad pathways of emotion regulation: one that relies on overt emotional control to reach a goal state, and another in which emotional recovery unfolds more naturally. This distinction parallels ecological and dynamical systems approaches to action (Turvey et al., 1978; Kelso et al., 1980; Haken et al., 1985), which emphasize that many complex systems—from motor behavior to physical phenomena—can settle into stable, adaptive states without continuous top-down control. Emotional processes may operate in a similar way, such that some emotional responses may benefit from being allowed to resolve on their own rather than being actively managed.

Consistent with this view, work on mindfulness-based acceptance suggests that releasing control can allow more adaptive or positive appraisals to emerge naturally over time (Garland et al., 2015; see also Segal et al., 2023). In a related sense, reappraisal itself may sometimes function as a gateway to letting go—particularly when reframing reduces the perceived need for continued emotional control (e.g., reframing negative emotions as “valuable” or “good”; Crum et al., 2020; Jamieson et al., 2012). At the same time, reappraisal preserving pieces of past feelings may not always be inherently maladaptive. Re-engaging such feelings can be functional, providing information that helps guide future behavior and decision making.

Taken together, these findings suggest that emotion regulation reflects a balance between authoring emotional experience and letting emotional stories write themselves. This perspective also aligns with regulatory flexibility frameworks (e.g., Bonanno, 2013; Bonanno and Burton, 2013), which emphasize that the effectiveness of any regulation strategy depends on its fit with situational demands rather than on the strategy itself. From this view, reappraisal and acceptance may represent distinct modes of regulatory engagement that become adaptive under different temporal and contextual constraints—control-based regulation supporting immediate action when emotional demands are high, and relinquishing control allowing emotional processes to reorganize more naturally. Rather than treating regulation as “strategies” that are uniformly adaptive or maladaptive, future work may benefit from conceptualizing emotion regulation as a dynamic interplay between emotional control and release—one that shifts across contexts, goals, and time as individuals adapt to emotionally meaningful experiences.

### Acceptance effects and measurement constraints

Given our focus on emotional control, an important limitation of the present study is that habitual acceptance was indexed through evaluative judgment of emotional experience rather than through direct measures of “letting go of” control (e.g., “I think some of my emotions are bad or inappropriate and I shouldn't feel them”; Baer et al., 2006). Although evaluative judgment and control are related, they are not equivalent. For example, in depressive states, negative self-judgment can coexist with reduced motivation or capacity to exert control (Gotlib and Joormann, 2010).

This distinction highlights a potential imprecision in how emotional control disengagement was operationalized. Whereas, habitual reappraisal was measured explicitly as a form of evaluative emotional control (e.g., “I control my emotions by changing the way I think”), the acceptance measure primarily captured nonjudgment rather than disengagement from regulatory goals. As such, the weaker moderation effect observed for acceptance may reflect limitations of measurement. Future research could benefit from assessing acceptance in ways that more directly capture control release, or from developing measures that better distinguish evaluative engagement from disengagement and assess them simultaneously. Nevertheless, the descriptive acceptance patterns were broadly consistent with the proposed framework and provide a preliminary basis for further investigation.

### Immediate affect vs. emotional reactivation

Another important pattern to note was the dissociation between participants' immediate emotional reactions to the divorce reminder and their later reports of self-relevant distress. Valence, arousal, sadness, and happiness after the film were uncorrelated with ratings of how much the reminder reactivated negative feelings tied to one's own divorce experience at the end of the study. This suggests that immediate affect may be a muddy or insensitive indicator of whether personally relevant negative emotions were reactivated (Levine and Safer, 2002), as momentary responses could reflect either empathizing with the characters or one's own experience. These findings highlight the importance of directly assessing self-referential emotional reactivation, rather than assuming it is reflected in immediate emotional intensity. At the same time, reminder-evoked distress relied on retrospective self-report, which may capture subjective meaning-making rather than implicit emotional activation alone. Future research could complement such measures with physiological indices of autonomic regulation (e.g., heart rate variability or related markers) to better distinguish consciously reported emotional experience from underlying regulatory dynamics.

In addition, although reappraisal reliably shaped action and movement dynamics, it did not significantly alter in-the-moment self-reported affect compared to no instruction or acceptance. While this diverges from typical patterns in studies using non-personally relevant inductions (e.g., Troy et al., 2018; Rompilla et al., 2022), it aligns with evidence that reappraisal can produce functional improvements in behavior even when subjective emotion does not shift (Rompilla et al., 2026; Zhu et al., 2023). However, small, nonsignificant trends did emerge in the expected direction (see Supplementary Figure S3), suggesting that the expected experiential effects may have reached significance with a larger sample.

### Limitations and constraints on generality

Several limitations qualify the present findings. First, the naturally occurring subsample was small, limiting statistical power and potentially attenuating moderation effects. Both significant and non-significant findings should therefore be interpreted as preliminary. Because the design was cross-sectional, the results reflect associations rather than changes over time, and we cannot determine whether reappraisal or acceptance shape emotional trajectories. Future work may benefit from alternative recruitment strategies—such as prescreening, multi-site sampling, or online data collection—to increase sample size, improve statistical power, and facilitate replication.

The sample consisted primarily of undergraduate students, a relatively homogeneous and WEIRD population (Henrich et al., 2010). In addition, the sample was predominantly female, and gender distribution differed across instruction conditions. Although gender was statistically controlled, these characteristics may limit generalizability and should be examined more systematically in future research. While the contrast between evaluative emotional control and disengagement from evaluative judgment may reflect broader regulatory principles, its applicability to more diverse or clinical populations remains uncertain.

Generalizability may also be constrained by the emotional context examined. The divorce-related film clip represented one form of past adversity, and emotional dynamics may differ for other experiences or emotions with distinct action tendencies (Frijda et al., 1989). Finally, regulation was assessed using brief laboratory instructions and retrospective self-report measures, which may not fully capture how reappraisal and acceptance unfold across repeated or real-world episodes.

Despite these constraints, the design also introduced a methodological advantage. Participants were not asked about parental divorce until after completing the task, increasing confidence that the film clip functioned as a personally meaningful yet unanticipated reminder rather than a targeted or anticipated manipulation.

## Conclusion

The present findings suggest that emotion regulation strategies differ not only in how they relate to immediate functioning, but also in how emotional responses to past adversity correspond with time since the event. Reappraisal was linked to efficient action and goal-directed control in the moment, yet habitual patterns of reappraisal were associated with different cross-sectional links between time since divorce and reminder-evoked distress. From an applied perspective, these results highlight the value of tailoring regulation strategies to situational demands rather than promoting any single approach as uniformly adaptive. In clinical or everyday contexts, encouraging flexible shifts between emotional-control-oriented strategies and forms of non-evaluative-acceptance of emotion may help individuals balance immediate functioning with longer-term emotional integration. As an exploratory proof-of-concept, the present work points toward a framework in which emotion regulation reflects a dynamic balance between evaluative control and non-evaluative openness to emotional experience—one that future longitudinal and confirmatory research can test more directly.

## Data Availability

The datasets presented in this study can be found in online repositories. The names of the repository/repositories and accession number(s) can be found below: https://osf.io/as2tq/files, in folder “Data_TheHiddenCostofControl.”
