# Supplementary material for: Action control in emotion regulation: when reappraisal may carry hidden costs
Source: Front Psychol. 2026 Apr 30;17:1790787. doi: 10.3389/fpsyg.2026.1790787 (PMC13173677; doi:10.3389/fpsyg.2026.1790787)
Supplement: Supplementary file 1 [file Table_1.docx]

*Appendix A*

**Acceptance**

Thank you for completing the second round of the Arrow game. For the next round, we will have you try to Accept your emotional responses to the film clips. There will be audio instructions on how to do this. Please listen (and read along) carefully.

According to Hayes et al. (2010), negative emotions are an important part of life and meaningful to experience fully. People who embrace their emotions tend to live happier, healthier, and more meaningful lives.

For this round, try to experience your feelings fully and do not try to control or change them in any way.

It is quite normal for this film clip to create some level of discomfort or negative emotion. Nevertheless, please let your feelings run their natural course and allow yourself to stay with your emotions, as fully as possible, without trying to control your feelings in any way.

In other words, try your best to fully accept the feelings that you experience during the film clip. This can be difficult at times, so it is very important that you try your best. Please ask the research assistant if you have questions about this task.

You will now begin Round 3 of 4. It is very important that you carefully watch the film clip, but allow your feelings to run their natural course without trying to control or change them. As you watch, take an Accepting attitude towards your emotions felt from the film clip.

**Reappraisal**

Thank you for completing the second round of the Arrow game. For the last round, we will have you try to control your emotional responses by changing your view of the film clip to be more Positive. There will be audio instructions on how to do this. Please listen (and read along) carefully.

According to Swanson et al. (2011), negative emotions are important to control and change when needed. People who can control their emotions tend to live happier, healthier, and more meaningful lives.

As you watch the film clip, try to think about the situation you see in a more Positive light.

You can achieve this in several different ways. For example, try to imagine advice that you could give to the characters in the film clip to make them feel better. This could be advice that would help them think about the positive bearing this event could have on their lives. Or, think about the good things they might learn from this experience.

Keep in mind that even though a situation may be painful in the moment, in the long run, it could make one’s life better, or have unexpected good outcomes.

In other words, try to think about the situation in as positive terms as you possibly can. This can be difficult at times, so it is very important that you try your best. Please ask the research assistant if you have questions about this task.

You will now begin Round 3 of 4. It is very important that you carefully watch the film clip, but think about it from a Positive perspective.

*References*

*(1) Instructions were adapted from:*

Troy, A. S., Shallcross, A. J., Brunner, A., Friedman, R., & Jones, M. C. (2018). Cognitive reappraisal and acceptance: Effects on emotion, physiology, and perceived cognitive costs. *Emotion, 18*(1), 58–74. [https://doi.org/10.1037/emo0000371](https://psycnet.apa.org/doi/10.1037/emo0000371)

*Appendix B*

Trajectories were imported into R mouse-tracking package, mousetrap (Kieslich & Henninger, 2017), from trial-wise x–y time series recorded in PsychoPy. To account for stimulus presentation lag on some trials (i.e., instances where the first x–y sample was recorded before the cursor had been reset to the bottom-center start position and the next trial appeared), the first recorded sample of each trajectory was overwritten with the fixed start position and trajectories were then start-aligned to this expected origin. Trials with fewer than 10 recorded samples were excluded. Response time outliers were removed using bounds of 0.3–5.0 s. After preprocessing, 99% of trials were preserved. Following the recommendations of Spivey et al. (2005), all trajectories were time-normalized to 101 equally spaced time steps before extracting measures.

On each trial, a color word (e.g., “Red”) appeared in a font color that either matched (e.g., “Red” in red ink) or conflicted with (e.g., “Red” in blue ink) its meaning. Participants were instructed to click the button corresponding to the font color, not the word meaning. Four response buttons were displayed across the top of the screen and labeled with the first letters of each color: “R,” “B,” “G,” and “Y” (i.e., Red, Blue, Green, Yellow). The order of these buttons remained fixed across blocks.

Each block consisted of 96 trials covering all 16 possible word–color combinations (12 incongruent and 4 congruent). Each unique combination appeared six times per block, resulting in 72 incongruent and 24 congruent trials. Each trial followed a consistent sequence: a 1-second fixation period, a 0.5-second target display, cursor-based response selection, and a 0.5-second progress bar before the next trial

Kieslich, P. J., & Henninger, F. (2017). Mousetrap: An integrated, open-source mouse-tracking package. *Behavior Research Methods*, *49*(5), 1652–1667. <https://doi.org/10.3758/s13428-017-0900-z>

Spivey, M. J., Grosjean, M., & Knoblich, G. (2005). Continuous attraction toward phonological competitors. *Psychological Science*, *16*(5), 367–374. <https://doi.org/10.1073/pnas.0503903102>

Table S1

| *Zero-Order Correlations Among Key Study Variables (N = 33)* | | | | | | | | | | | | | | |
| --- | --- | --- | --- | --- | --- | --- | --- | --- | --- | --- | --- | --- | --- | --- |
| **Key Variables** | **1** | **2** | **3** | **4** | **5** | **6** | **7** | **8** | **9** | **10** | **11** | **12** | **13** | **14** |
| 1. Divorce Recollection | — |  |  |  |  |  |  |  |  |  |  |  |  |  |
| 2. Divorce Distance | -.33† | — |  |  |  |  |  |  |  |  |  |  |  |  |
| 3. Habitual Reappraisal (ERQ) | .08 | .28 | — |  |  |  |  |  |  |  |  |  |  |  |
| 4. Habitual Acceptance | .18 | −.08 | -.24 | — |  |  |  |  |  |  |  |  |  |  |
| 5. Valence (Δ) | .07 | .09 | -.27 | .01 | — |  |  |  |  |  |  |  |  |  |
| 6. Arousal (Δ) | .11 | .21 | -.14 | .18 | .36* | — |  |  |  |  |  |  |  |  |
| 7. Sadness (Δ) | -.01 | -.17 | -.13 | .16 | -.59** | -.15 | — |  |  |  |  |  |  |  |
| 8. Happiness (Δ) | .10 | -.15 | -.16 | .04 | .38* | .11 | -.41* | — |  |  |  |  |  |  |
| 9. Response Time (Δ) | -.26 | -.05 | .00 | -.05 | -.07 | -.27 | -.04 | .12 | — |  |  |  |  |  |
| 10. Time of Max Velocity (Δ) | -.17 | -.15 | -.13 | .10 | -.10 | -.18 | -.01 | .22 | .86** | — |  |  |  |  |
| 11. Time of Max Acceleration (Δ) | -.18 | -.18 | -.04 | .05 | -.18 | -.11 | .12 | .12 | .88** | .88** | — |  |  |  |
| 12. Idle Time (Δ) | -.16 | -.09 | -.26 | .09 | .13 | -.23 | -.03 | .25 | .75** | .81** | .72** | — |  |  |
| 13. Accuracy (Δ) | -.10 | -.00 | .21 | -.07 | -.15 | -.08 | .07 | -.15 | .17 | .04 | .12 | -.06 | — |  |
| 14. Divorce Exposure (Numeric) | .29 | .04 | -.08 | -.04 | .14 | .20 | .29 | -.26 | -.42* | -.39* | -.37* | -.26 | -.14 | — |
| 15. Divorce Exposure Block | .20 | .01 | -.23 | -.16 | .02 | .15 | .28 | -.36* | -.07 | -.08 | -.02 | -.04 | .03 | .75** |
| *Note.* Values are Pearson correlation coefficients. Δ indicates change scores relative to baseline (Block 1). *p*† < .10. *p** < .05. *p*** < .01. *p**** < .001. | | | | | | | | | | | | | | |

Table S2

*Baseline characteristics by instruction condition for participants with parental divorce.*

| Variable *M* (*SD*) | No Instruction | Acceptance | Reappraisal | *p* value |
| --- | --- | --- | --- | --- |
| *N* | 14 | 8 | 11 |  |
| Age | 20.00 (2.29) | 21.38 (7.25) | 19.27 (0.90) | .505 |
| Divorce distance (years) | 10.71 (5.37) | 10.62 (7.27) | 11.27 (5.73) | .964 |
| Habitual reappraisal | 5.14 (1.09) | 4.69 (0.95) | 4.97 (1.17) | .643 |
| Habitual acceptance | 3.04 (0.75) | 3.00 (0.99) | 3.12 (1.07) | .953 |
| Variable *N* (*proportion*) | No Instruction | Acceptance | Reappraisal | *p* value |
| Gender: f | 8 (57.1%) | 7 (87.5%) | 9 (81.8%) | .294 |
| Gender: m | 6 (42.9%) | 1 (12.5%) | 2 (18.2%) | -- |

*Note: p*-values are for one-way ANOVA comparing means between no instruction, acceptance, and reappraisal. Gender proportions were assessed using Fisher’s exact test. Cell sizes for male were too small to assess.

| Table S3  *Means and SD for Stroop performance* | | | |
| --- | --- | --- | --- |
| Measure *M* (*SD*) | No Instruction (n = 14) | Acceptance (n = 8) | Reappraisal (n = 12) |
| Response Time | −0.13 (0.17) | −0.09 (0.15) | −0.31 (0.19) |
| Idle Time | −0.11 (0.14) | −0.10 (0.05) | −0.22 (0.20) |
| Time of Max Velocity | −0.06 (0.14) | −0.04 (0.18) | −0.18 (0.15) |
| Time of Max Acceleration | −0.09 (0.12) | −0.02 (0.16) | −0.20 (0.15) |
| Accuracy | −0.01 (0.02) | 0.00 (0.01) | −0.02 (0.06) |

*Note.* Means represent baseline-corrected values (subtracting the neutral block [Block 1] from performance at the assigned regulation condition).

| Table S4  *Linear Mixed-Effects Model Results for Stroop Performance Measures with Trial-Level Data* | | | | | |
| --- | --- | --- | --- | --- | --- |
| DV | Predictor | *b* | *SE* | *t* | *p* |
| **RT** | **StrategyNoInstruction** | 0.124 | 0.053 | 2.33 | **.025*** |
|  | **StrategyAcceptance** | 0.082 | 0.036 | 2.27 | **.027*** |
|  | CongruencyIncongruent | 0.056 | 0.016 | 3.56 | <.001*** |
|  | baseline_RT_log_c | 0.700 | 0.090 | 7.80 | <.001*** |
|  | Genderm | -0.004 | 0.028 | -0.16 | .878 |
|  | DivExposure_Block | 0.005 | 0.032 | 0.15 | .885 |
|  | StrategyNoInstruction × CongruencyIncongruent | -0.026 | 0.021 | -1.22 | .221 |
|  | StrategyAcceptance × CongruencyIncongruent | -0.027 | 0.024 | -1.11 | .269 |
| DV | Predictor | *b* | *SE* | *t* | *p* |
| **Idle Time** | **StrategyNoInstruction** | -0.013 | 0.053 | -0.24 | .814 |
|  | **StrategyAcceptance** | 0.064 | 0.035 | 1.82 | **.076**† |
|  | CongruencyIncongruent | 0.002 | 0.010 | 0.16 | .874 |
|  | baseline_idle_log_c | 0.528 | 0.134 | 3.93 | <.001*** |
|  | Genderm | 0.003 | 0.029 | 0.12 | .909 |
|  | DivExposure_Block | -0.050 | 0.033 | -1.49 | .147 |
|  | StrategyNoInstruction × CongruencyIncongruent | 0.014 | 0.014 | 1.01 | .311 |
|  | StrategyAcceptance × CongruencyIncongruent | -0.016 | 0.016 | -1.01 | .311 |
| DV | Predictor | *b* | *SE* | *t* | *p* |
| **Time of Max Velocity** | **StrategyNoInstruction** | 0.085 | 0.038 | 2.26 | **.028*** |
|  | **StrategyAcceptance** | 0.078 | 0.029 | 2.66 | **.009**** |
|  | CongruencyIncongruent | 0.052 | 0.018 | 2.95 | .003** |
|  | baseline_vel_log_c | 0.436 | 0.118 | 3.71 | <.001*** |
|  | Genderm | 0.046 | 0.019 | 2.49 | .018* |
|  | DivExposure_Block | 0.017 | 0.021 | 0.80 | .429 |
|  | StrategyNoInstruction × CongruencyIncongruent | -0.027 | 0.023 | -1.16 | .246 |
|  | StrategyAcceptance × CongruencyIncongruent | -0.028 | 0.027 | -1.03 | .301 |
| DV | Predictor | *b* | *SE* | *t* | *p* |
| **Time of Max Acceleration** | **StrategyNoInstruction** | 0.110 | 0.082 | 1.35 | .185 |
|  | **StrategyAcceptance** | 0.146 | 0.058 | 2.53 | **.014*** |
|  | CongruencyIncongruent | 0.060 | 0.028 | 2.15 | .032* |
|  | baseline_acc_log_c | 0.847 | 0.125 | 6.80 | <.001*** |
|  | Genderm | -0.058 | 0.042 | -1.38 | .178 |
|  | DivExposure_Block | -0.024 | 0.049 | -0.49 | .626 |
|  | StrategyNoInstruction × CongruencyIncongruent | -0.013 | 0.037 | -0.34 | .731 |
|  | StrategyAcceptance × CongruencyIncongruent | -0.023 | 0.043 | -0.53 | .594 |
| DV | Predictor | *b* | *SE* | *t* | *p* |
| **Accuracy** | StrategyNoInstruction | -0.013 | 0.053 | -0.24 | .814 |
|  | **StrategyAcceptance** | 0.064 | 0.035 | 1.82 | **.076**† |
|  | CongruencyIncongruent | 0.002 | 0.010 | 0.16 | .874 |
|  | baseline_idle_log_c | 0.528 | 0.134 | 3.93 | <.001*** |
|  | Genderm | 0.003 | 0.029 | 0.12 | .909 |
|  | DivExposure_Block | -0.050 | 0.033 | -1.49 | .147 |
|  | StrategyNoInstruction × CongruencyIncongruent | 0.014 | 0.014 | 1.01 | .311 |
|  | StrategyAcceptance × CongruencyIncongruent | -0.016 | 0.016 | -1.01 | .311 |
| ***Note.*** Linear mixed-effects models were estimated in R using the lmer() function from the lme4 package. Models were specified as DV ~ Strategy × Congruency + baseline covariate + Gender + DivExposure_Block + (1 \| Participant) and fit at the trial level, with random intercepts for participants to account for within-subject variability. Fixed effects included strategy (reappraisal, no instruction, acceptance), congruency (congruent, incongruent), and their interaction, along with covariates for baseline performance (Block 1, grand-mean centered), gender, and the block in which the divorce reminder occurred. Strategy was coded with reappraisal as the reference condition, and congruency with congruent trials as the reference. Continuous dependent variables (response time, time of maximum velocity, time of maximum acceleration, and idle time) were log-transformed prior to analysis to reduce skewness; when necessary, a constant was added prior to transformation to accommodate zero or negative values. Because accuracy was binary at the trial level, it was analyzed using a generalized mixed-effects model with a binomial distribution and logit link, with baseline accuracy represented using an empirical logit transformation of Block 1 accuracy. Models were fit using maximum likelihood estimation (REML = FALSE for linear models), and fixed effects were evaluated using Type III tests via lmerTest. Follow-up comparisons were conducted using estimated marginal means (emmeans) with Holm correction.  Across all dependent measures, the pattern of fixed effects was consistent with the primary ANCOVA analyses. Congruency effects were observed for response time and movement dynamics, and strategy effects showed similar directional differences relative to the reference condition. No significant Strategy × Congruency interactions emerged. These findings indicate that the observed effects are robust to analytic approach and are not driven by aggregation at the between-subject level. †p < .10, *p < .05, **p < .01, ***p < .001. | | | | | |

Table S5

*Instructed Emotion Regulation and Emotional Experience*

| Emotional Measure | *F*(2, 28) | *p* | Partial η² |
| --- | --- | --- | --- |
| Valence | 1.54 | .233 | .099 |
| Arousal | 0.39 | .682 | .027 |
| Sadness | 0.50 | .611 | .035 |
| Happiness | 0.39 | .684 | .027 |

*Note.* Values reflect between-subjects ANCOVAs testing the effect of instruction condition (reappraisal, acceptance, no instruction) on emotional experience reported immediately after the divorce reminder, controlling for gender and exposure block. Emotional responses were baseline-corrected by subtracting ratings following the neutral block (Block 1).

Table S6

*Moderation Model Predicting Divorce Reminder–Evoked Distress by Divorce Distance and Habitual Reappraisal*

| Predictor | *b* | *SE* | *t* | *p* | LLCI | ULCI |
| --- | --- | --- | --- | --- | --- | --- |
| Intercept | 6.09 | 2.07 | 2.94 | .007 | 1.83 | 10.36 |
| Divorce Distance (Dist) | −0.46 | 0.16 | −2.79 | .010 | −0.80 | −0.12 |
| Habitual Reappraisal | −0.64 | 0.45 | −1.44 | .162 | −1.56 | 0.27 |
| Dist × Habitual Reappraisal | 0.08 | 0.03 | 2.30 | .030 | 0.01 | 0.15 |
| DivExposure: No Instruction | −0.45 | 0.53 | −0.84 | .406 | −1.54 | 0.64 |
| DivExposure: Reappraisal | 0.08 | 0.52 | 0.16 | .877 | −1.00 | 1.16 |
| Gender (male = 1, female = 0) | −0.01 | 0.47 | −0.02 | .983 | −0.99 | 0.97 |

*Note.* Coefficients reflect an ordinary least squares moderation model (PROCESS Model 1). Dist = divorce distance (years since parental divorce); _1 = interaction term between divorce distance and the moderator (habitual reappraisal in Table S5; habitual acceptance in Table S6); DivExposure = instruction condition during the divorce reminder (reference group = Acceptance); Gender coded as 0 = female and 1 = male. Coefficients (b) are unstandardized. SE = standard error; LLCI and ULCI = lower and upper bounds of the 95% confidence interval.

Table S7

*Moderation Model Predicting Divorce Reminder–Evoked Distress by Divorce Distance and Habitual Acceptance*

| Predictor | *b* | *SE* | *t* | *p* | LLCI | ULCI |
| --- | --- | --- | --- | --- | --- | --- |
| Intercept | 1.48 | 1.96 | 0.75 | .458 | −2.55 | 5.51 |
| Divorce Distance (Dist) | 0.14 | 0.17 | 0.81 | .424 | −0.22 | 0.50 |
| Habitual Acceptance | 0.39 | 0.55 | 0.72 | .478 | −0.73 | 1.52 |
| Dist × Habitual Acceptance | −0.07 | 0.05 | −1.26 | .219 | −0.18 | 0.04 |
| DivExposure: No Instruction | −0.28 | 0.60 | −0.47 | .643 | −1.52 | 0.95 |
| DivExposure: Reappraisal | 0.86 | 0.69 | 1.26 | .220 | −0.55 | 2.27 |
| Gender (male = 1, female = 0) | 0.12 | 0.51 | 0.24 | .812 | −0.92 | 1.16 |

*Note.* Coefficients reflect an ordinary least squares moderation model (PROCESS Model 1). Dist = divorce distance (years since parental divorce); Int_1 = interaction term between divorce distance and the moderator (habitual reappraisal in Table S5; habitual acceptance in Table S6); DivExposure = instruction condition during the divorce reminder (reference group = Acceptance); Gender coded as 0 = female and 1 = male. Coefficients (b) are unstandardized. SE = standard error; LLCI and ULCI = lower and upper bounds of the 95% confidence interval.

Figure S1.

*Example of Stroop trials*

*
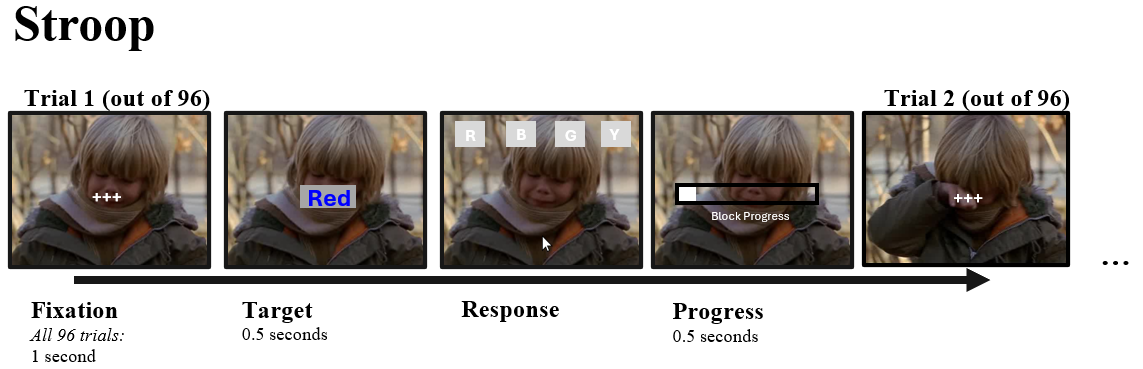
*

*Note. Each block consisted of 96 trials covering all 16 possible word–color combinations (12 incongruent and 4 congruent). Each unique combination appeared six times per block, resulting in 72 incongruent and 24 congruent trials. Each trial followed a consistent sequence: a 1-second fixation period, a 0.5-second target display, cursor-based response selection, and a 0.5-second progress bar before the next trial*

Figure S2

*Distribution of divorce distance
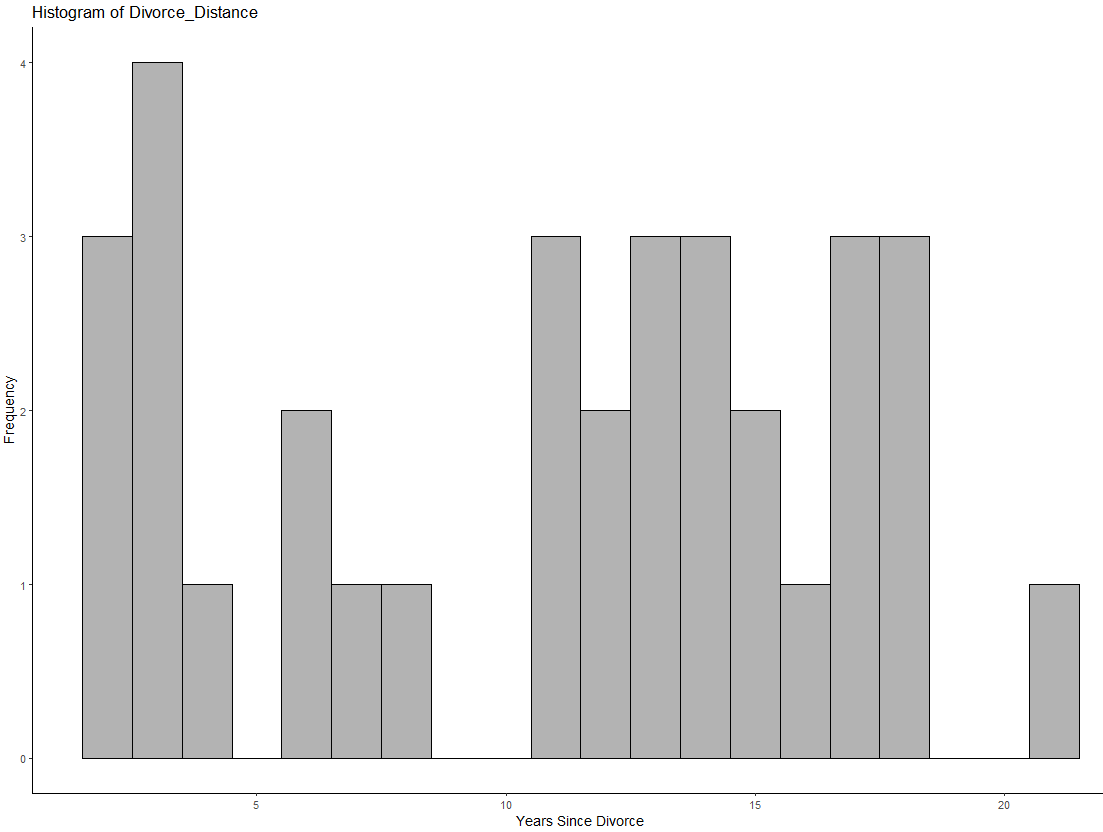
*

*
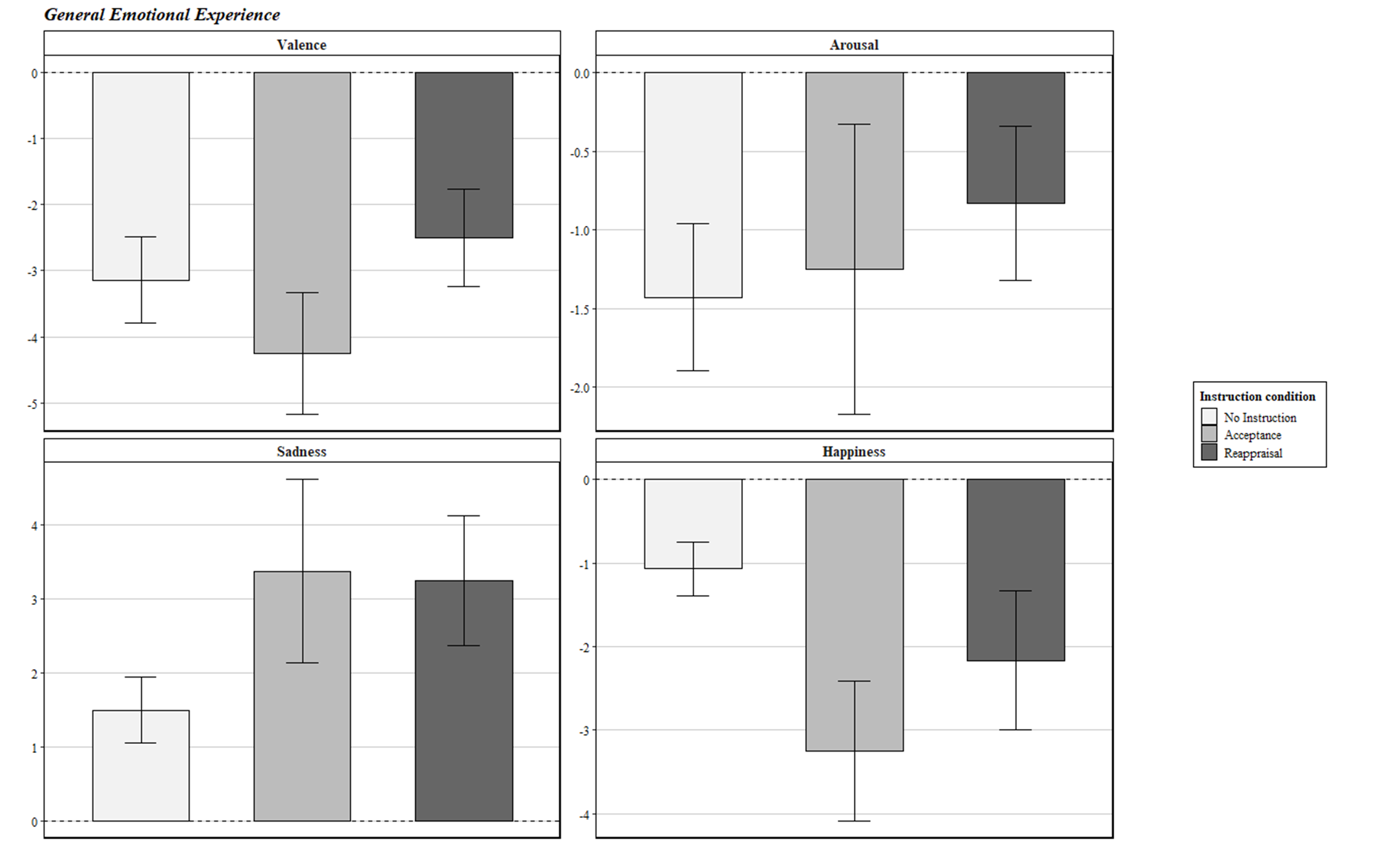
*Figure S3

*Note.* Bars represent change scores calculated by subtracting baseline performance in Block 1 (neutral film) from performance during the divorce-reminder block. Negative values indicate less reported emotion relative to baseline. Error bars represent ±1 SE. Asterisks in panel titles indicate a significant main effect of instruction condition.

Figure S4.

*Johnson-Neyman Habitual Reappraisal Moderation*

*
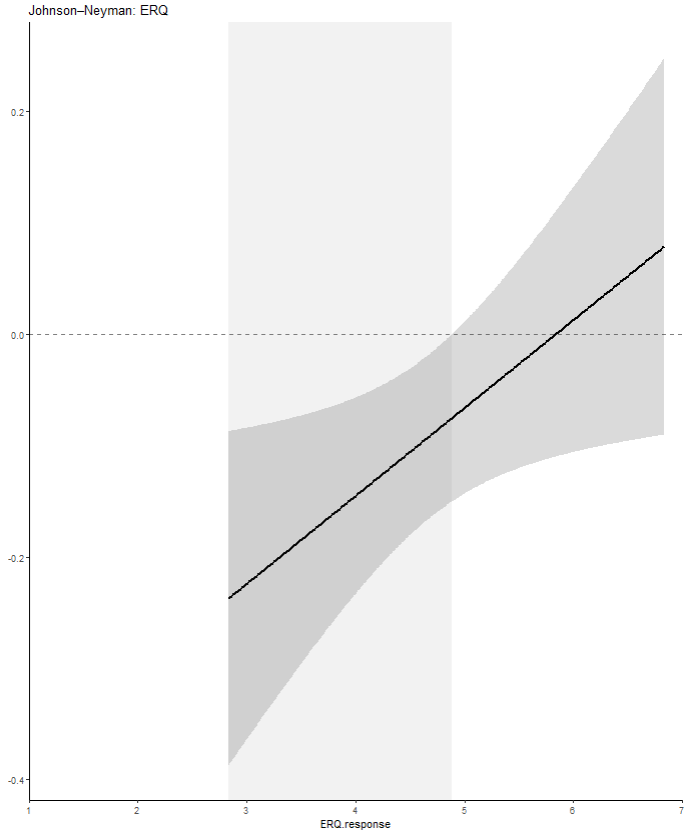
*

Figure S5.

*Johnson-Neyman Habitual Acceptance Moderation*

*
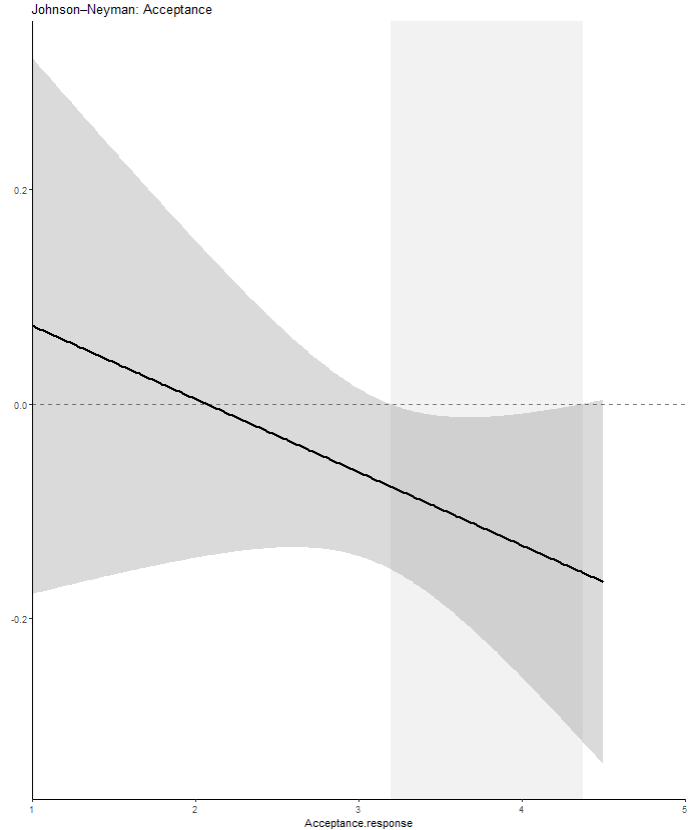
*

Figure S6

*Habitual Reappraisal Moderation Residual, Q–Q, and scale–location plots*

*
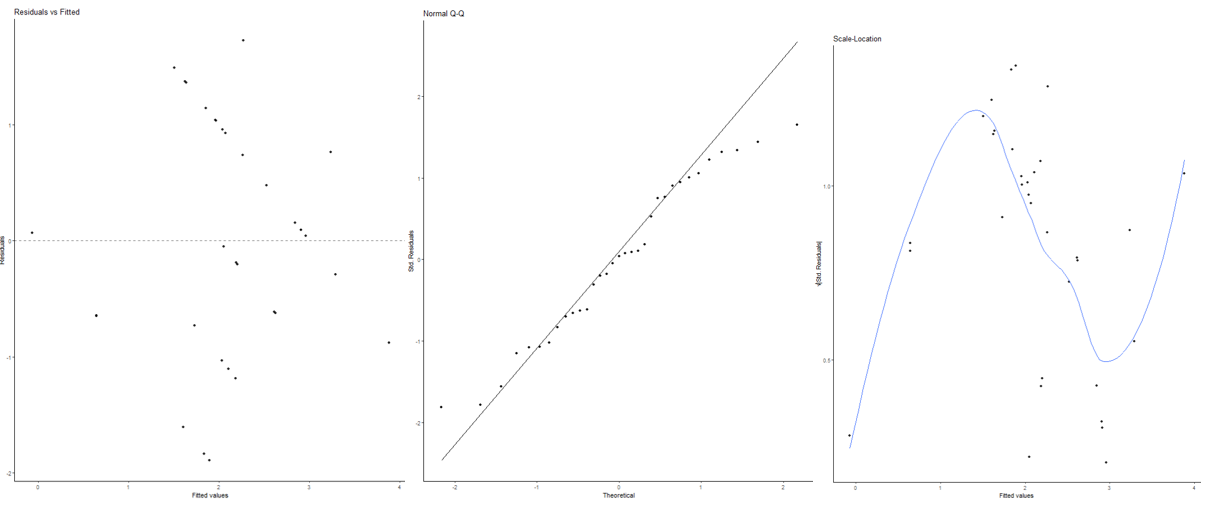
*

Figure S7

*Habitual Acceptance Moderation Residual, Q–Q, and scale–location plots*

*
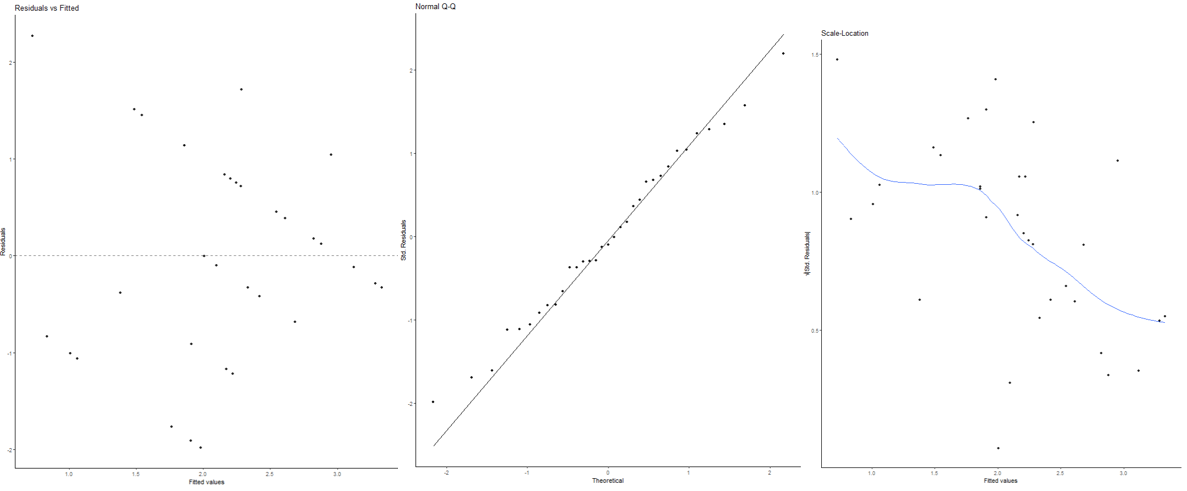
*
